# Novel Role for the AnxA1-Fpr2/ALX Signaling Axis as a Key Regulator of Platelet Function to Promote Resolution of Inflammation

**DOI:** 10.1161/CIRCULATIONAHA.118.039345

**Published:** 2019-06-03

**Authors:** Elena Y. Senchenkova, Junaid Ansari, Felix Becker, Shantel A. Vital, Zaki Al-Yafeai, Erica M. Sparkenbaugh, Rafal Pawlinski, Karen Y. Stokes, Jennifer L. Carroll, Ana-Maria Dragoi, Cheng Xue Qin, Rebecca H. Ritchie, Hai Sun, Hugo H. Cuellar-Saenz, Mara R. Rubinstein, Yiping W. Han, A. Wayne Orr, Mauro Perretti, D. Neil Granger, Felicity N.E. Gavins

**Affiliations:** 1 Departments of Molecular and Cellular Physiology (E.Y.S., J.A., S.A.V., K.Y.S., D.N.G., F.N.E.G.); 2 Pathology and Translational Pathobiology (Z.A.-Y., A.W.O.); 3 Neurosurgery (H.S., H.H.C.-Z.); 4 Cellular Biology and Anatomy (A.W.O.); 5 INLET (J.L.C., A.-M.D.); 6 Feist–Weiller Cancer Center (J.L.C., A.-M.D.), Louisiana State University Health Sciences Center–Shreveport.; 7 Department for General, Visceral, and Transplant Surgery, University Hospital Muenster, Germany (F.B., H.S.).; 8 Department of Medicine, University North Carolina Chapel Hill (E.M.S., R.P.).; 9 Heart Failure Pharmacology Laboratory, Baker Heart and Diabetes Institute, Melbourne, Victoria, Australia (C.X.Q., R.H.R.).; 10 Division of Periodontics, College of Dental Medicine (M.R.R., Y.W.H.), Columbia University, New York.; 11 Department of Microbiology and Immunology, Vagelos College of Physicians and Surgeons (Y.W.H.), Columbia University, New York.; 12 William Harvey Research Institute, Queen Mary University of London, UK (M.P.).; 13 Department of Life Sciences, Brunel University London, Uxbridge, Middlesex, UK (F.N.E.G.).

**Keywords:** annexin A1, formyl peptide receptor, inflammation, integrins, stroke, thrombosis

## Abstract

**Background:**

Ischemia reperfusion injury (I/RI) is a common complication of cardiovascular diseases. Resolution of detrimental I/RI-generated prothrombotic and proinflammatory responses is essential to restore homeostasis. Platelets play a crucial part in the integration of thrombosis and inflammation. Their role as participants in the resolution of thromboinflammation is underappreciated; therefore we used pharmacological and genetic approaches, coupled with murine and clinical samples, to uncover key concepts underlying this role.

**Methods:**

Middle cerebral artery occlusion with reperfusion was performed in wild-type or annexin A1 (AnxA1) knockout (AnxA1^−/−^) mice. Fluorescence intravital microscopy was used to visualize cellular trafficking and to monitor light/dye–induced thrombosis. The mice were treated with vehicle, AnxA1 (3.3 mg/kg), WRW4 (1.8 mg/kg), or all 3, and the effect of AnxA1 was determined in vivo and in vitro.

**Results:**

Intravital microscopy revealed heightened platelet adherence and aggregate formation post I/RI, which were further exacerbated in AnxA1^−/−^ mice. AnxA1 administration regulated platelet function directly (eg, via reducing thromboxane B_2_ and modulating phosphatidylserine expression) to promote cerebral protection post-I/RI and act as an effective preventative strategy for stroke by reducing platelet activation, aggregate formation, and cerebral thrombosis, a prerequisite for ischemic stroke. To translate these findings into a clinical setting, we show that AnxA1 plasma levels are reduced in human and murine stroke and that AnxA1 is able to act on human platelets, suppressing classic thrombin-induced inside-out signaling events (eg, Akt activation, intracellular calcium release, and Ras-associated protein 1 [Rap1] expression) to decrease α_IIb_β_3_ activation without altering its surface expression. AnxA1 also selectively modifies cell surface determinants (eg, phosphatidylserine) to promote platelet phagocytosis by neutrophils, thereby driving active resolution. (n=5–13 mice/group or 7–10 humans/group.)

**Conclusions:**

AnxA1 affords protection by altering the platelet phenotype in cerebral I/RI from propathogenic to regulatory and reducing the propensity for platelets to aggregate and cause thrombosis by affecting integrin (α_IIb_β_3_) activation, a previously unknown phenomenon. Thus, our data reveal a novel multifaceted role for AnxA1 to act both as a therapeutic and a prophylactic drug via its ability to promote endogenous proresolving, antithromboinflammatory circuits in cerebral I/RI. Collectively, these results further advance our knowledge and understanding in the field of platelet and resolution biology.

## Abstract 2

Supplemental Digital Content is available in the text.

**Clinical Perspective**

**What Is New?**

- The findings of lower plasma levels of the anti-inflammatory/proresolving protein annexin A1 (AnxA1) among patients with acute ischemic stroke may help to explain the exacerbated thromboinflammatory response observed in ischemia reperfusion injury.
- Administration of AnxA1 promotes cerebral protection against thromboinflammation and development of subsequent thrombotic events after stroke.
- AnxA1 is able to reduce platelet activation and thrombosis via the suppression of integrins (the main platelet receptors involved in platelet adhesion and aggregation).

**What Are the Clinical Implications?**

- Our findings regarding the multifaceted role that AnxA1 plays in stroke may provide a means to extend the intervention window for both medical and surgical therapeutic modalities and make the intervention safer for patients.
- Furthermore, the protective effect of AnxA1 may improve the outcome of stroke intervention by limiting the development of thrombotic events during and after the intervention.
- Our data could pave the way for novel and more personalized approaches to antiplatelet therapy.

Inflammation and thrombosis play crucial roles in the pathophysiological cascade of cardiovascular diseases, especially in ischemic stroke.^1^ The interdependent connection of both processes in eliciting a detrimental prothrombotic and proinflammatory state after ischemia reperfusion injury (I/RI) has led to the concept of thromboinflammation.^1–4^ The rapid inflammatory response that develops during reperfusion leads to subsequent cerebral microvascular dysfunction with platelets adhering at ischemic vascular lesions, thereby increasing the risk of secondary thrombotic events and further tissue injury.^5^

Administration of agents that ameliorate the aggressive thromboinflammatory state rapidly after ischemic insults represent attractive novel treatment strategies for I/RI.^1–3^ Although peripheral inflammatory cells such as neutrophils and monocytes have been implicated in the resolution of thromboinflammation, the role that platelets may play in this process is largely undefined thus far.

The active process of resolution involves a tightly orchestrated series of highly regulated biochemical mediators,^6–8^ such as endogenous annexin A1 (AnxA1) and lipoxin A_4_.^9^ In humans, the biological effects of AnxA1 and its mimetic peptides are mediated through 3 G-protein–coupled receptors, termed formyl peptide receptors (FPR1, FPR2, FPR3).^10^ Although the protective effects of AnxA1 have been well characterized in different disease models,^6–12^ only a few studies (with mixed outcomes) have focused on the effect(s) of AnxA1 (previously termed Lipocortin 1) on platelet function, with a particular emphasis on the of AnxA1 in the regulation of intracellular phospholipase A2.^13–20^ Thus, we wanted to further investigate the effect(s) of AnxA1 on platelet function (eg, platelet adhesion, activation, aggregation, and thrombosis) in the context of the resolution of thromboinflammation.

Previously, our findings elucidated that targeting lipoxin A_4_ receptor Fpr2/ALX (murine orthologue to human FPR2/ALX) inhibits cerebral inflammation in a focal transient I/RI model.^9^ However, we and others have yet to discern the effect(s) of AnxA1 on platelets and whether strategies focused on the AnxA1-Fpr2/ALX axis may substantially add to the development of new treatment possibilities for I/RI. We were therefore interested in closing this knowledge gap.

Herein, we found a novel modulatory role of AnxA1 on platelets. Specifically, AnxA1 administration reduced both platelet adherence to the inflamed cerebral endothelium after stroke and regulated the state of platelet activation. AnxA1 moderated platelet aggregation (by reducing platelet–platelet aggregate formation), diminished the prothrombotic potential of platelets (reflected in decreased thromboxane B_2_ production and reduced phosphatidylserine [PS] expression), and suppressed active integrin α_IIb_β_3_ levels, thereby reducing the risk of thrombotic events. We also present novel data regarding the ability of AnxA1 to promote the phagocytosis of platelets by neutrophils. Finally, we report a previously unknown role for AnxA1 as a preventative strategy both for cerebral I/RI (by its ability to reduce cerebral thrombosis, a prerequisite for stroke) and for subsequent thrombotic events after I/RI.

## Methods

A detailed Methods section is provided in the online-only Data Supplement. The data that support the findings of this study are available from the corresponding author on reasonable request.

### Animals

Animal experiments complied with ARRIVE (Animal Research: Reporting In Vivo Experiments) guidelines and followed the European Union Directive (2010/63/EU) or LSUHSC-S IACUC. Wild-type (WT) C57BL/6 mice or AnxA1^−/−^ mice^21^ were used.

### Human Samples

The study was approved by the institutional review board of the LSUHSC-S (STUDY00000572 and STUDY00000261) and conducted in accordance with the Declaration of Helsinki. Written informed consent was obtained from the participants.

### Receptor Agonists and Drug Treatment

Vehicle (saline), whole protein AnxA1 (3.3 mg/kg,^21^ Cambridge Research Biochemicals, Cleveland, UK), and WRW4 (1.8 mg/kg,^22^ Tocris, Bristol, UK) were administered intravenously at the start of cerebral reperfusion.

### AnxA1 Quantification in Plasma

Human or murine AnxA1 ELISA kits (MyBioSource) were used to quantify the plasma levels of AnxA1 (see online-only Data Supplement).

### Transient Focal Middle Cerebral Artery Occlusion With Reperfusion

Transient focal middle cerebral artery occlusion with reperfusion (tMCAo/R) was performed for 60 min followed by 4 or 24 h of reperfusion according to standard operating procedure in our laboratory.^9^ Sham animals were subject to the same operative procedure without occlusion.

### Platelet and Leukocyte Labeling

Platelets were isolated from donor mice, ex vivo fluorescently labeled with carboxyfluorescein succinimidyl ester (90 µmol/L, 10 min, Sigma–Aldrich, St Louis, MO), and injected into recipient mice, followed by 0.02% rhodamine 6G (Sigma–Aldrich) to label circulating leukocytes in vivo.^9^

#### Neutrophil Depletion

Neutropenia was induced using mouse antineutrophil serum (1A8; BioXCell, West Lebanon, NH; 150 μg/mouse).^23^

#### Neutrophil Isolation and Adoptive Transfer

Neutrophils were isolated, labeled with CellTracker^24^ (ThermoFisher, Waltham, MA), and treated with WRW4 (10 μmol/L, 10 min, Tocris) before injection into recipient neutropenic tMCAo/R mice. The mice were then treated with AnxA1 (see online-only Data Supplement).^20^

### Cerebral Intravital Fluorescence Microscopy

Intravital microscopy was performed according to standard operating procedure in our laboratory using a Zeiss Axioskop microscope (Zeiss, New York, NY; see online-only Data Supplement).^9^

### Confocal Microscopy

To visualize endothelial platelet–neutrophil aggregate (PNA) formation in vivo, the mice were injected with specific antibodies to label: neutrophils (eFluor 488 [green]–labeled anti-mouse Ly-6G, 2 μg/mouse; eBioscience, San Diego, CA) and platelets (Dylight 649 [red]-labeled antimouse CD42, 1 μg/mouse; Emfret Analytics, Eibelstadt, Germany; see online-only Data Supplement).^9^

### Systemic PNA Assessment

Systemic PNAs were assessed by flow cytometry in blood from tMCAo/R mice treated with either vehicle or AnxA1. Leukocytes were labeled with rat antimouse CD45.2–FITC, Gr-1–PE, and F4/80–eFluor450 and isotype controls (eBioscience), and platelets were labeled with CD41-APC (see online-only Data Supplement).^21^

### Assessment of Activated Platelets by Flow Cytometry

Two-color staining of activated murine and human α_IIb_β_3_, cell surface CD41a, P-selectin, and AnxAV (to measure PS) was performed using flow cytometry (see online-only Data Supplement).^26^

### Platelet Aggregation Assay

Platelet-rich plasma was freshly collected from tMCAo/R mice treated with either vehicle or AnxA1 and used to monitor platelet aggregation velocity after agonist exposure using a laser-particle analyzer (Lumex Ltd., St. Petersburg, Russia; see online-only Data Supplement).^27^

### Thrombosis

Thrombosis in cerebral vessels was induced using the light/dye thrombosis model (see online-only Data Supplement).^25^ Thirty minutes before onset of thrombosis, the mice were treated with vehicle or AnxA1 (1 µg/mouse).

### Ras-Associated Protein 1 Activity

Ras-associated protein 1 (Rap1) activity assay was performed according to instructions from the supplier (see online-only Data Supplement).

### Western Blotting

Freshly prepared platelets (1×10^7^ cells) were incubated with AnxA1 (100 ng/1×10^6^) platelets for 15 min followed by thrombin stimulation (0.1 U) for 3 min. Platelet pellets were prepared for western blotting for Akt and FPR2 (see online-only Data Supplement).

### Measurement of Intracellular Ca^2+^ Levels in Fluo-3-Acetoxymethyl Ester

Washed platelets (1×10^6^) were stained with fluo-3-acetoxymethyl ester (5 μmol/L) for 30 min at 37°C as previously described,^28^ and some samples were incubated with vehicle or AnxA1 (100 ng) before thrombin stimulation. Fluorescent intensity of fluo-3-acetoxymethyl ester–loaded platelets was immediately recorded using BD LSR II as previously described (see online-only Data Supplement).^28^

### Phagocytosis Assay

Neutrophil phagocytosis of platelets was performed using the IncuCyte ZOOM (Essen BioScience, Inc., Ann Arbor, MI; see online-only Data Supplement).

### Cytokines in Plasma and Brain Tissue

After 24 h of reperfusion, plasma and brain hemisphere homogenates were obtained. The levels of pro- and anti-inflammatory cytokines and thromboxane B_2_ were measured using standard ELISAs (see online-only Data Supplement).

### Infarct Volume

After 24 h of reperfusion, the brains were removed and stained with 2% 2,3,5-triphenyltetrazolium chloride (Sigma–Aldrich). Sections were photographed, and digitized images of each brain section (and the infarcted area) were quantified using National Institutes of Health 1.57 Image software.^9^

### Neurological Score

A 5-point neurological deficit score was used: 0 indicates no deficit; 1, failure to extend right paw; 2, circling to the right; 3, falling to the right; and 4, unable to walk spontaneously.^9^ The mice were evaluated at 24 h of reperfusion.

### Blood–Brain Barrier Permeability

Blood–brain barrier (BBB) permeability was assessed using Evans blue extravasation.^9^ BBB permeability was normalized by dividing tissue Evans blue concentration (micrograms per gram of brain weight) by plasma concentration (micrograms per milliliter).

### Thrombin–Antithrombin Complex Measurement

Plasma was collected from WT and AnxA1^−/−^ mice, and thrombin–antithrombin was measured using thrombin–antithrombin complex ELISAs (Assay Pro, Saint Charles, MO).

### D-Dimer

Plasma was collected from WT and AnxA1^−/−^ mice, and D-dimer was measured using an Asserachrom D-dimer kit (no. 00947; Diagnostica Stago, Parsippany, NJ).

### Statistical Analysis

All data were tested to follow a normal distribution using a Kolmogorov–Smirnov test of normality with a Dallal–Wilkinson–Lillie for the corrected *p* value. The data that passed the normality assumption was analyzed using Student *t* test (2 groups) or ANOVA with Bonferroni post-tests (more than 2 groups). The data that failed the normality assumption were analyzed using the nonparametric Mann–Whitney U test (2 groups) or Kruskal–Wallis with Dunn’s test (more than 2 groups). Analysis was performed using Graph Pad Prism5 software (San Diego, CA). The data are shown as mean values ± SEM or median with interquartile range (neurological score only). The differences were considered statistically significant at a value of *P*<0.05.

## Results

### Mice Lacking AnxA1 Possess Augmented Platelet Responses Coupled With Increased Cerebral Damage After I/RI

**Figure 1.**
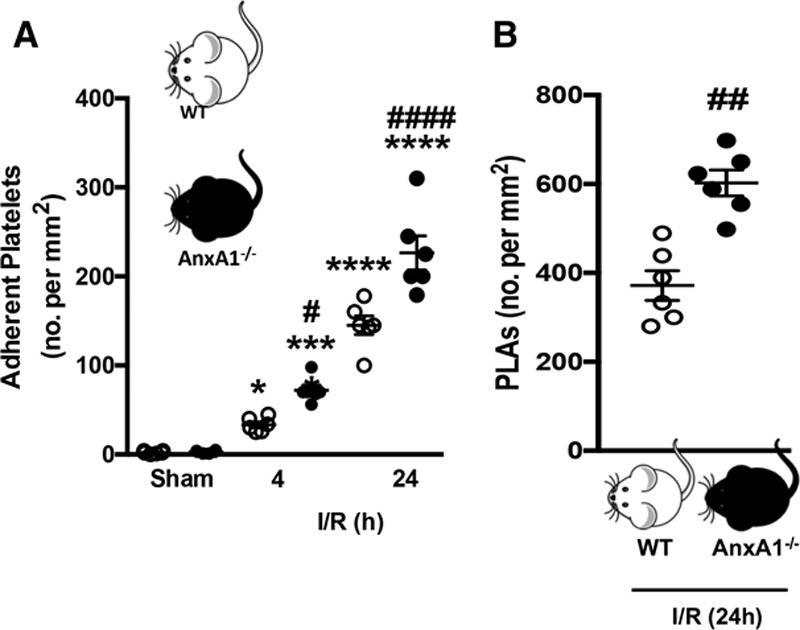
**Platelet and platelet–leukocyte interactions in the cerebral microcirculation are heightened in annexin A1 knockout (AnxA1^−/−^) mice after ischemia reperfusion injury (I/R).** Wild-type (WT) and AnxA1^−/−^ mice were subjected to transient middle cerebral artery occlusion for 60 min, followed by 4- or 24-h reperfusion. Intravital fluorescence microscopy was performed to assess cellular interactions in the cerebral microcirculation (pial vessels) of mice subjected to cerebral I/R. Platelets were labeled with carboxyfluorescein succinimidyl ester (90 µmol/L), and leukocytes were labeled with rhodamine 6G (0.02%). Platelet interactions were quantified in terms of numbers (no.) of adherent platelets on the endothelium (**A**, cells stationary for ≥2 s) and platelets interacting directly with adherent leukocytes on the endothelium (**B**), termed platelet–leukocyte aggregates (PLAs). The data are means±SEM of 6 mice per group with 2 to 3 vessels per mouse and assessed by ANOVA with Bonferroni post hoc test (**A**) or Mann–Whitney test (**B**). *P<0.05, ***P<0.001, and ****P<0.0001 vs sham control of the same genotype. #*P*<0.05, ##*P*<0.01, and ####*P*<0.0001 vs a different genotype at the same time point.

To determine whether a deletion of *AnxA1* impacts platelet recruitment and the I/RI-elicited inflammatory response and to extend the macroscopic severity of cerebral I/RI, we first performed 60-min tMCAo followed by 4 or 24 h reperfusion in AnxA1^−/−^ mice. AnxA1^−/−^ mice displayed a significant increase in platelet adhesion to the cerebral endothelium at both time points after reperfusion (Figure 1A). This was coupled to heightened platelet-–leukocyte aggregate formation (Figure 1B) and leukocyte adhesion (Figure IA in the online-only Data Supplement). These observations were not related to changes in hemodynamic parameters because no differences among mouse genotypes were detected (Table I in the online-only Data Supplement). Furthermore, the effects on platelets were also paralleled with increased stroke severity as displayed by increased neurological score, infarct volume, and BBB permeability in both the contralateral and ipsilateral hemispheres of tMCAo/R AnxA1^−/−^ mice (Figure IC through IE in the online-only Data Supplement), suggesting that deletion of the *AnxA1* gene is associated with increased neurovascular inflammation after cerebral I/RI. The *AnxA1* gene deletion is also not associated with spontaneous thrombosis and bleeding because plasma levels of both thrombin–antithrombin III complex (an indicator of thrombin production and accurately reflects the activation of coagulation)^29,30^ and D-dimer (an indicator of ongoing fibrinolysis)^30,31^ were similar to their WT counterparts (Figure II in the online-only Data Supplement).

In line with the exacerbated cerebral inflammatory state, plasma cytokine levels of interleukin (IL)-1β, TNFα, and IL-6 were all increased in AnxA1^−/−^ mice compared with WT mice under the same conditions. There were no differences found in CCL2 or IL-10 levels (Table 1). Local cytokine levels were all significantly elevated in the left (injured) hemisphere of WT and AnxA1^−/−^ mice after I/RI (Table 1). Interestingly, AnxA1^−/−^ mice also displayed higher IL-10 levels in the left hemisphere versus the right, suggesting a possible compensatory mechanism. However, both TNFα and IL-1β levels were elevated in AnxA1^−/−^ mice compared with WT mice. No cytokine was above control values in the contralateral hemisphere of either WT or AnxA1^−/−^ mice, suggesting that these inflammatory mechanisms are confined within the ischemic region (ie, ipsilateral side).

**Table 1.**
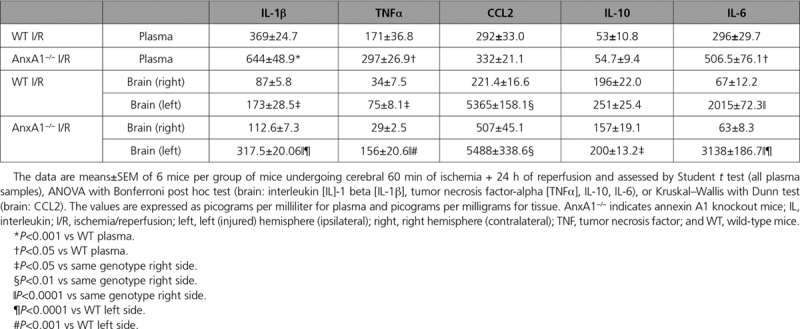
Cytokine Profiles Are Augmented in AnxA1^−/−^ Mice Ischemia Reperfusion Injury

**Figure 2.**
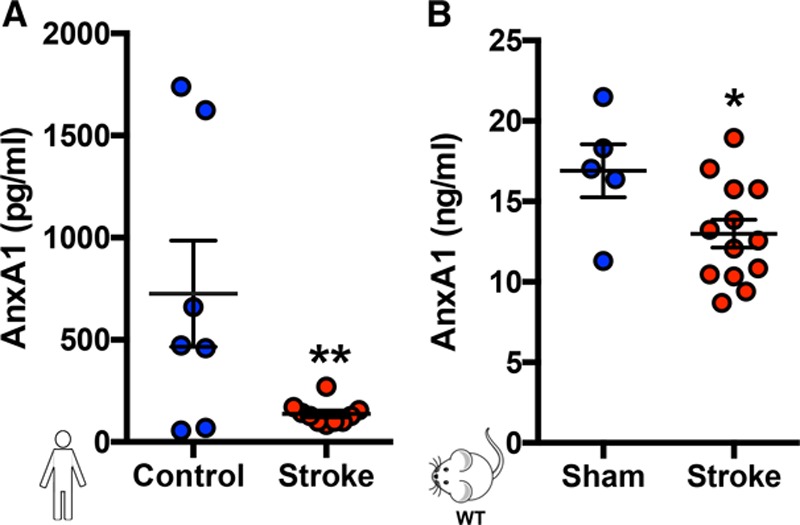
**Plasma levels of AnxA1 are reduced in human and murine stroke.** Plasma was collected from control volunteers and stroke patients (**A**) and mice with and without stroke (**B**, transient middle cerebral artery occlusion for 60 min followed by 24 h of reperfusion). Annexin A1 (AnxA1) levels were assessed by ELISA. The data are means±SEM of 7 to 13, with 10 humans per group (8 samples were collected from control volunteers, but 1 outlier [defined as at least 2 standard deviations] was removed; 11 samples were collected from stroke patients, but 1 outlier was removed) and 5 to 13 mice per group and assessed by a Student *t* test (**A** and **B**). ^*^P<0.05 and ^**^P<0.01 vs control. WT indicates wild type.

Plasma levels of AnxA1 were found to be lower in stroke patients (Table 2) versus the control group (Figure 2A). This reduced AnxA1 level was also mirrored in plasma samples obtained from WT mice subjected to tMCAo for 60 min followed by 24 h of reperfusion (Figure 2B), supporting the translation of our findings to a clinical setting.

**Table 2.**
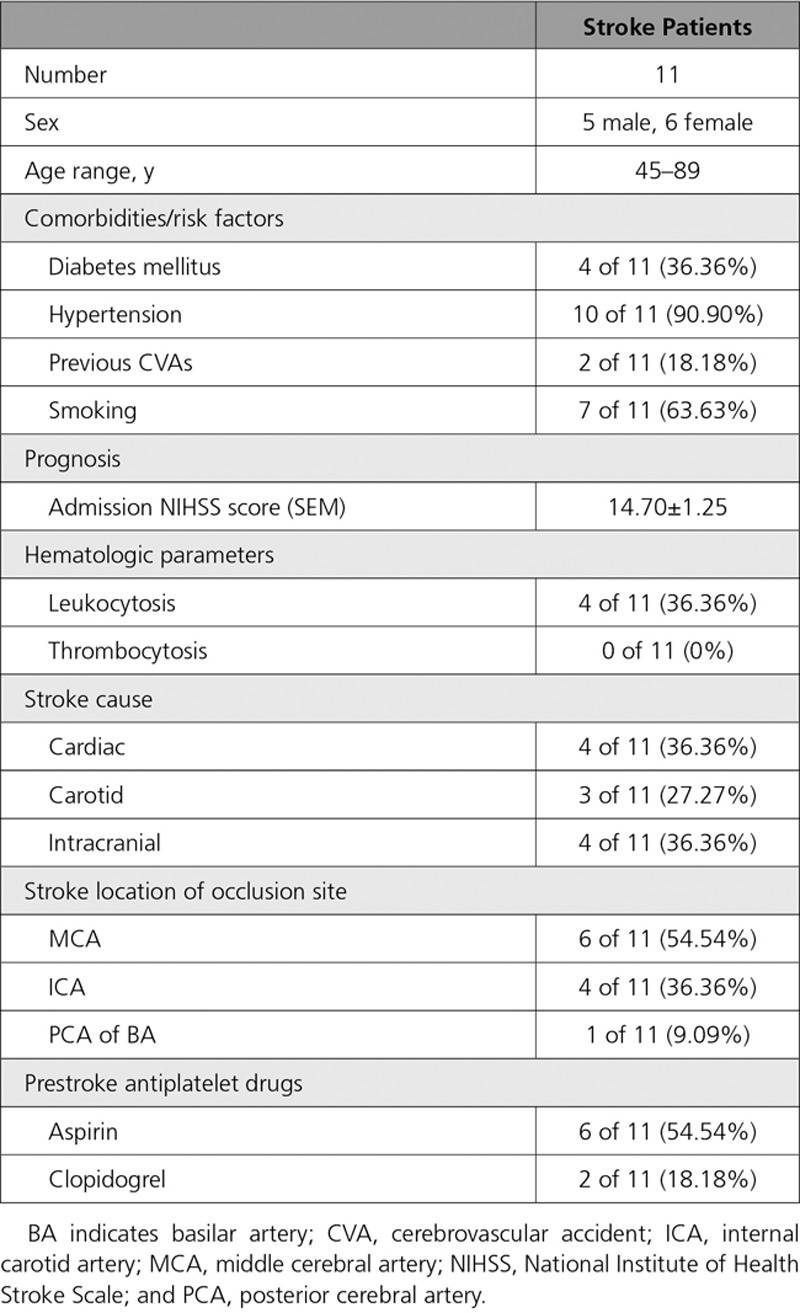
Demographic and Clinical Characteristics of Stroke Patients

### Blocking Fpr2/ALX Inhibits the Effect of AnxA1 on Ischemia-Induced Platelet Interactions

**Figure 3.**
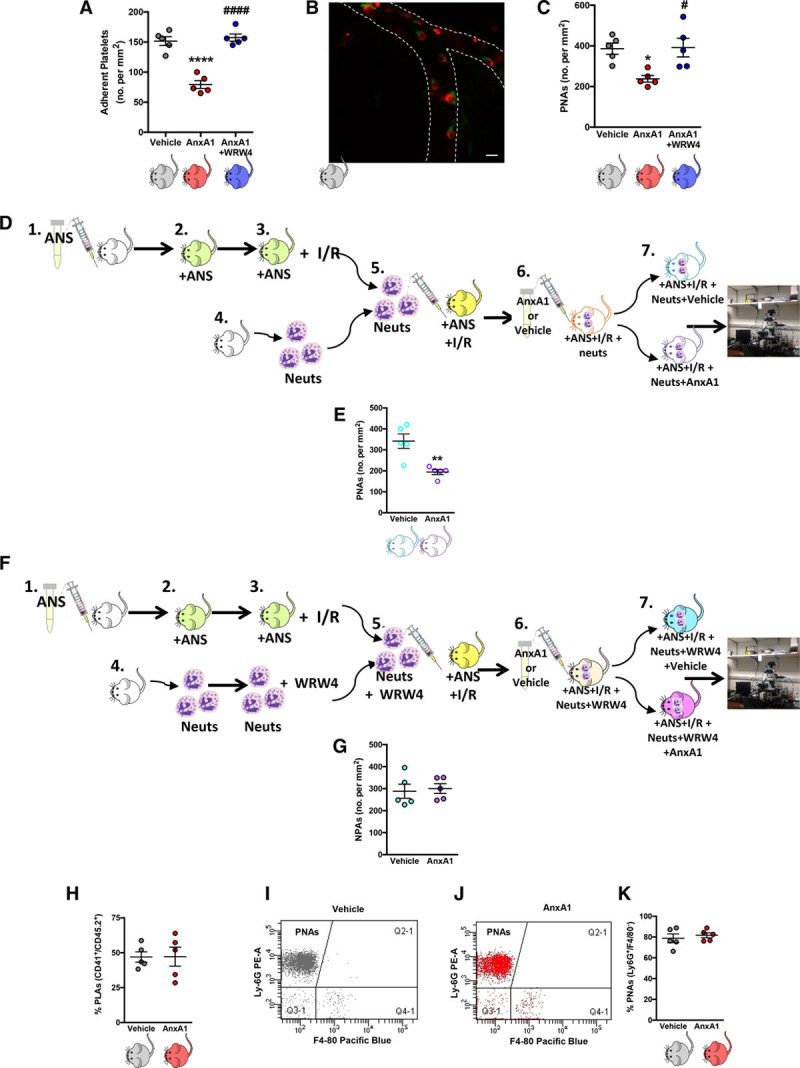
**Administration of exogenous annexin A1 (AnxA1) moderates platelet interactions in the brain microcirculation after ischemia reperfusion injury (I/R).** Wild-type mice were subjected to transient middle cerebral artery occlusion for 60 min, followed by 24 h of reperfusion (tMCAo/R). **A**, Vehicle, AnxA1 (3.3 mg/kg), or WRW4 (1.8 mg/kg) were administered intravenously at the start of reperfusion and numbers (no.) of platelet adhesion (cells stationary for at least 2 s) were quantified. **B**, Representative image of cerebral platelet-neutrophil aggregates (PNAs) 24 h after reperfusion as measured by confocal intravital microscopy. Neutrophils (Neuts, **arrow**) were labeled with eFluor 488 (green)-labeled anti-mouse Ly-6G, 2 μg/mouse. Platelets (arrowhead) were labeled with Dylight 649 (red)-labeled antimouse CD42, 1 μg/mouse. The scale bar indicates 20 μm. **C**, Quantification of endothelial-PNAs in the cerebral microcirculation. **D**, Adoptive transfer experiments were performed by injecting neutrophils isolated from donor mice into neutropenic recipient mice. In steps 1 and 2, recipient mice were rendered neutropenic by administration of antineutrophil serum (ANS). In step 3, neutropenic mice were subjected to cerebral I/R by tMCAo/R. In step 4, neutrophils were isolated from donor mice. In step 5, neutrophils were injected into neutropenic recipient mice subjected MCAo/R. In step 6, the mice were then treated with vehicle (saline) or AnxA1 (3.3 mg/kg) for 30 min before intravital microscopy, which is shown in step 7. **E**, Quantification of PNAs in the cerebral microcirculation from **D** using intravital microscopy. **F**, Adoptive transfer experiments were performed by injecting neutrophils isolated from donor mice into neutropenic recipient mice. In steps 1 and 2, recipient mice were rendered neutropenic by administration of ANS. In step 3, neutropenic mice were subjected to cerebral I/R by tMCAo/R. In step 6, the mice were then treated with vehicle (saline) or AnxA1 (3.3 mg/kg) for 30 min before intravital microscopy, which is shown in step 7. **G**, Quantification of NPAs (neutrophil platelet aggregates) in the cerebral microcirculation from **F** using intravital microscopy. **H** through **K**, Blood samples were taken from wild-type mice subjected to MCAo, followed by a 24-h reperfusion and treatment with vehicle (saline) or AnxA1 and analyzed by flow cytometry to assess systemic aggregate formation: quantification of circulating. **H**, Platelet–leukocyte aggregates (PLAs, CD45.2^+^, and CD41^+^). **I** and **J**, Flow cytometry population groups of PNAs, which are denoted within the CD45.2^+^, CD41^+^ population as Ly-6G^+^, and F4/80^−^ aggregates. **K**, Quantification of circulating PNAs. The data are means±SEM of 5 mice/group and assessed by ANOVA with a Bonferroni post hoc test (**A** and **B**), Mann–Whitney test (**E**), or a Student *t* test (**G**, **H**, and **K**). *P<0.05 and ****P<0.0001 vs vehicle (saline)–treated control. #*P*<0.05 and ####*P*<0.0001 vs AnxA1.

We sought to determine whether AnxA1 administration could alter platelet–endothelial and platelet–leukocyte interactions. Figure 3 shows that administration of AnxA1 to WT mice subjected to tMCAo decreases both intravascular platelet adhesion (Figure 3A) and PNA accumulation (Figure 3B and 3C) on the cerebral endothelium when compared with saline-injected animals. In addition, these protective effects produced by AnxA1 were translated in reduced neurological score (assessed 24 h after reperfusion), which correlated with decreased infarct volumes (data not shown).

The importance of Fpr2/ALX as the key receptor in mediating effects of AnxA1 has been widely studied.^8–12,19–22^ However, this has not been shown in the context of platelet function. We found that when coadministered with AnxA1, the selective Fpr2/ALX antagonist WRW4^11^ was able to successfully abrogate the protective actions of AnxA1 on platelet and PNA adhesion to the cerebral endothelium (Figure 3A and 3C). These data further support the growing body of evidence that the protective effects of AnxA1 are mediated through Fpr2/ALX and identify a previously unknown effect of the AnxA1 Fpr2/ALX pathway to mitigate the thromboinflammatory effects of platelets.

### AnxA1 Reduces PNA Recruitment via Fpr2/ALX

Next we tested whether AnxA1 would inhibit endothelial PNA recruitment via a direct action on neutrophil Fpr2/ALX, as reported for another agonist of this receptor, aspirin-triggered lipoxin A_4_.^7^ To test this hypothesis, isolated neutrophils were infused into neutropenic mice after tMCAo, and these mice were subsequently treated with AnxA1 or saline vehicle (Figure 3D). This treatment resulted in reduced cerebral endothelial PNA formation (Figure 3E). To further confirm that the observed effects were specifically due to the actions of AnxA1 on Fpr2/ALX, we repeated the experiments in presence of the Fpr2/ALX specific antagonist WRW4. To this end, isolated neutrophils were pretreated with WRW4 and then infused into neutropenic mice after tMCAo. Animals were subsequently treated with AnxA1 or saline vehicle (Figure 3F). These experiments showed that selective antagonism of neutrophil Fpr2/ALX prevented the ability of AnxA1 to reduce PNA recruitment to the cerebral endothelium after stroke (Figure 3G). We then further tested whether these results were specific to local PNA recruitment to the damaged cerebral endothelium or whether AnxA1 might have an additional effect on systemic aggregate formation after cerebral I/RI. Interestingly, we found that AnxA1 did not affect the percentage of circulating PNAs within the circulating platelet–leukocyte aggregate population in the blood of mice subjected to tMCAo for 60 min followed by 24 h of reperfusion (Figure 3H through 3K and Figures III and IV in the online-only Data Supplement). These intriguing results indicate that although AnxA1 reduces endothelial PNA formation in the brain, it is not due to a decrease in the number of circulating PNAs.

### AnxA1 Inhibits the Prothrombotic Activity of Platelets Post-I/RI

**Figure 4.**
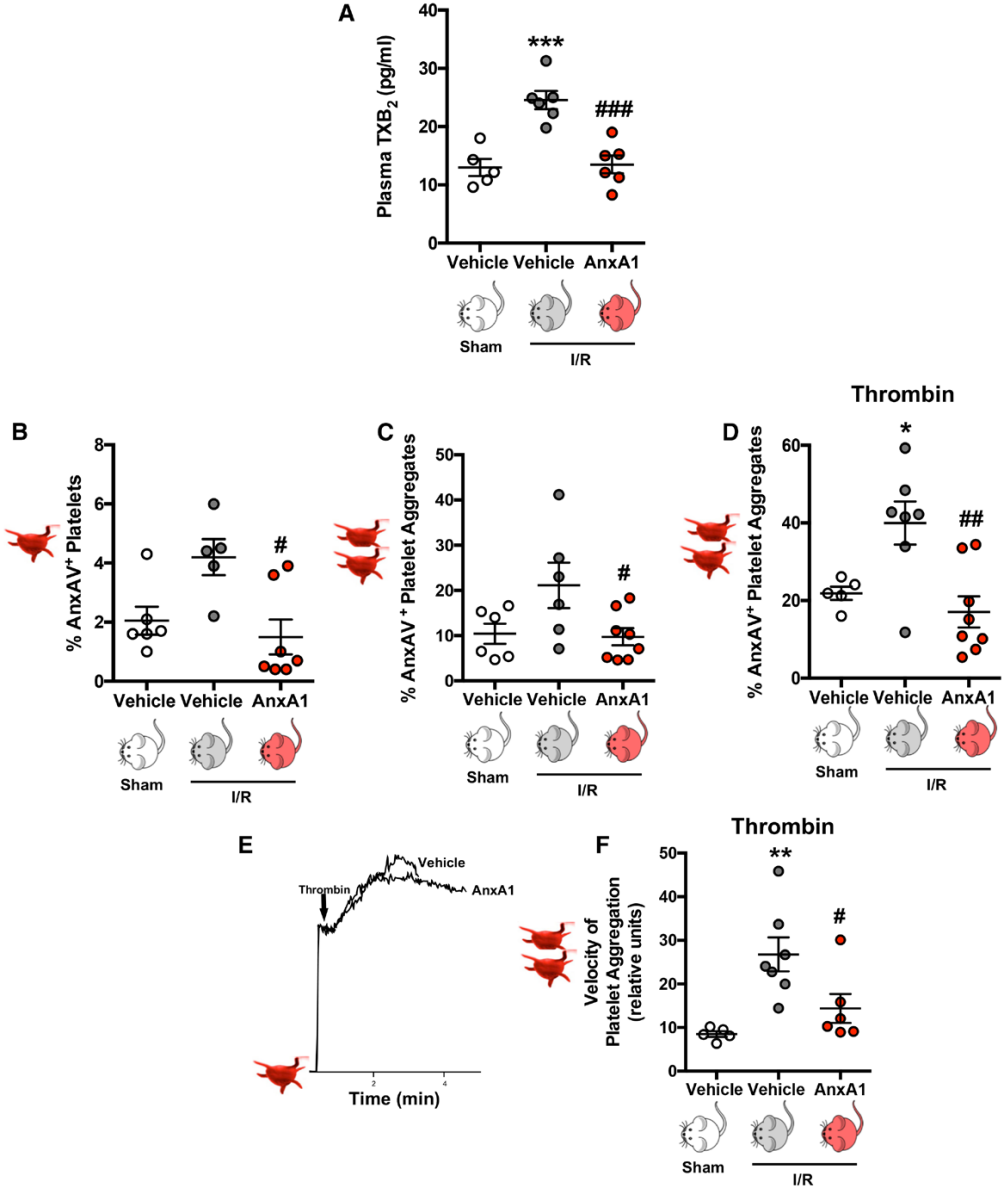
**Annexin A1 (AnxA1) reduces platelet activation/aggregation postischemia reperfusion injury (I/R).** Wild-type mice were subjected to sham or transient middle cerebral artery occlusion for 60 min, followed by 24 h of reperfusion. Vehicle (saline) or AnxA1 (3.3 mg/kg) was administered intravenously at the start of reperfusion. **A**, At the end of reperfusion, plasma thromboxane B_2_ (TXB_2_) levels were measured. **B** through **F**, Platelets were isolated for quantification as follows. **B** through **D**, Using flow cytometry, phosphatidylserine externalization (using annexin AV [AnxAV] staining, presented as a percentage of AnxAV-positive platelets) was quantified on single platelets (**B**), platelet–platelet aggregates (**C**), or platelet–platelet aggregates after further stimulation with thrombin (**D**, 0.1 U/mL). **E**, Representative platelet aggregation velocity chart in response to thrombin (0.1 U/mL). **F**, Velocity of aggregate formation was measured using a low-angle light-scattering technique. The data are means±SEM of 5 to 8 mice per group and assessed by ANOVA with Bonferroni post hoc test (**A** through **D** and **F**). *P<0.05, **P<0.01, and ***P<0.001 vs platelets from vehicle treated sham mice. #*P*<0.05, ##*P*<0.001, and ###*P*<0.001 vs platelets from vehicle-treated I/R mice.

Having provided clear evidence that deletion of *AnxA1* is associated with increased platelet and PNA adherence after stroke (Figure 1) and administration of AnxA1 ameliorates the detrimental effects of I/RI by diminishing platelet interactions at the cerebral endothelium, we wanted to evaluate the contribution of AnxA1 on platelet activation/aggregation after cerebral I/RI. We first measured platelet activation by quantifying plasma levels of the thromboxane A_2_ stable metabolite thromboxane B_2_. Figure 4A shows that mice treated with AnxA1 have decreased thromboxane B_2_ levels in comparison with those levels observed in vehicle (saline)–treated tMCAo/R mice. Platelets facilitate blood coagulation by externalizing PS on their cell surface, and PS exposure is directly related to the procoagulant activity of activated platelets.^33^ Figure 4B shows that AnxA1 decreased PS exposure (expressed as AnxAV^+^) on single platelets after tMCAo/R (determined by flow cytometry), as well as on platelet–platelet aggregates (Figure 4C). Interestingly, this effect of AnxA1 on platelet PS exposure was also present when platelets were further ex vivo stimulated with either thrombin or ADP (Figure 4D and Figure VA in the online-only Data Supplement).

### AnxA1 Reduces the Velocity at Which Platelets Aggregate After Cerebral I/RI

Having shown that AnxA1 reduces the prothrombotic activity of platelets from tMCAo/R mice, we next addressed whether this was related to the ability of platelets to form aggregates. Figure 4E and 4F shows that AnxA1-treated tMCAo/R mice exhibited decreased velocity of platelet aggregation to thrombin (and ADP; Figure VB and VC in the online-only Data Supplement). These data provide further evidence that not only is AnxA1 able to reduce the prothrombotic potential of platelets, but it is also able to reduce the velocity at which platelets form platelet–platelet aggregates, thereby reducing the risk of thrombosis, eg, subsequent thrombotic events in stroke.

### AnxA1 Thwarts Thrombosis and Secondary Thrombotic Events Poststroke

**Figure 5.**
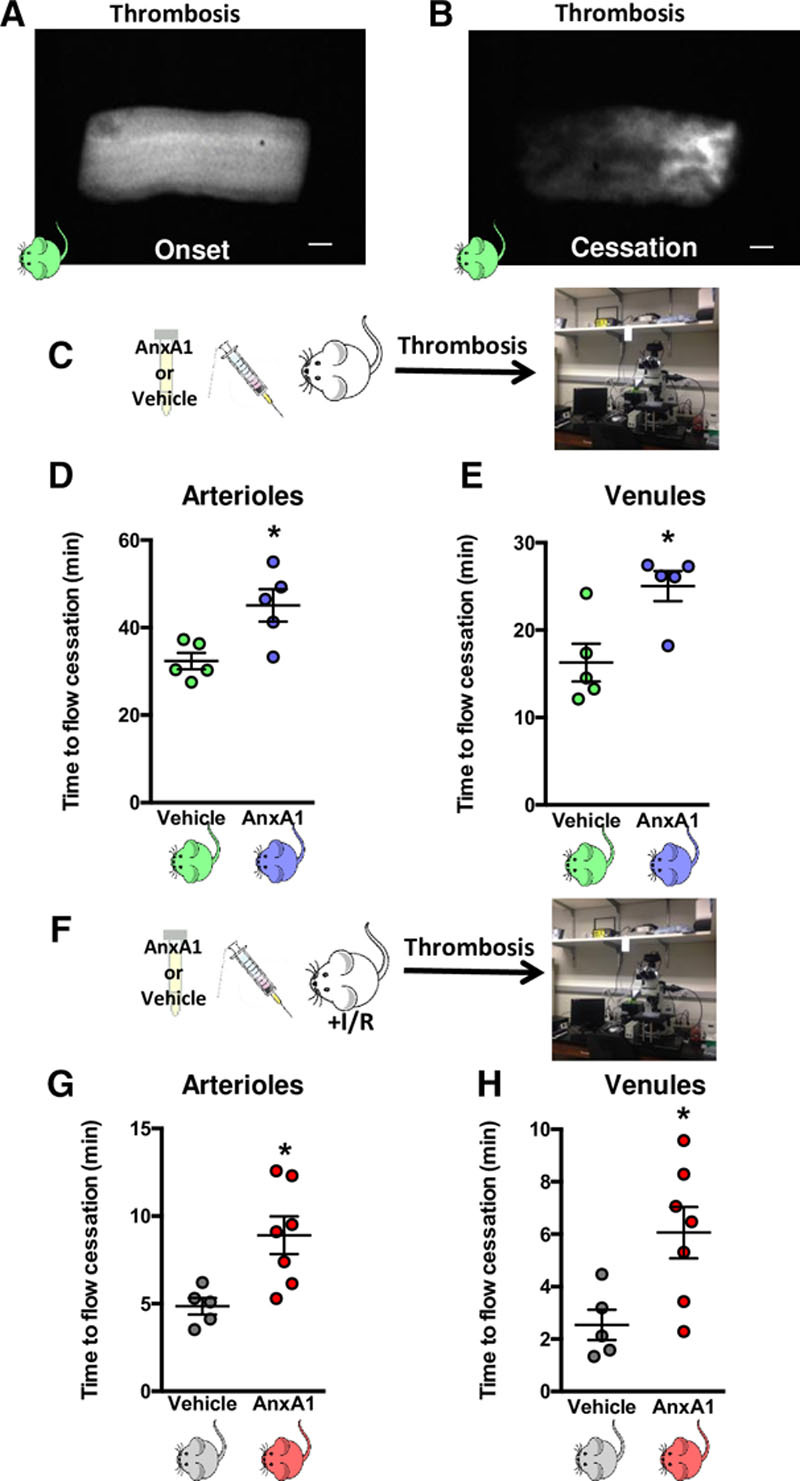
**Annexin A1 (AnxA1) protects against initial cerebral thrombosis and development of subsequent thrombotic events. A** and **B**, Still images of cerebral pial vessels showing onset (**A**) and cessation (**B**) of blood flow in the light/dye-induced thrombus model in wild-type (WT) mice. **C** through **E**, Effects of AnxA1 (**C**, 1 µg/mouse) or saline vehicle injected 20 min before the onset of thrombus formation in cerebral arterioles (**D**) and venules (**E**). **F**, The mice were subjected to transient middle cerebral artery occlusion for 60 min, followed by 24 h of reperfusion. **G** and **H**, At the end of reperfusion, the mice were treated with vehicle (saline) or AnxA1 (1 µg/mouse) 20 min before the onset of light/dye-induced thrombus formation in cerebral arterioles (**G**) and venules (**H**). The data are means±SEM of 5 to 7 mice/group and assessed by a Student *t* test (**D**, **E**, **G**, and **H**). *P<0.05 vs vehicle treated control. The scale bar indicates 10 μm. I/R indicates ischemia reperfusion injury.

Having assessed the novel anti-inflammatory and antithrombotic effects of AnxA1 on platelets isolated from AnxA1-treated mice after cerebral I/RI, we wanted to address in vivo whether AnxA1 administration might also be effective in reducing the prothrombotic events that may ultimately lead to stroke and reducing the effect of subsequent thrombotic events after stroke. Figure 5A through 5E shows that AnxA1 is able to prolong blood flow cessation time in both cerebral arterioles and venules, suggesting that AnxA1 is able to modify the thromboinflammatory environment, which may lead to ischemic stroke. Given the impact of subsequent thrombotic events in stroke, we finally assessed whether AnxA1 had a similar impact on thrombus formation in mice after cerebral I/RI. Thus, we set up an animal model combining tMCAo with 24 h of reperfusion, followed by the light/dye method to measure cerebral blood flow cessation time in both cerebral arterioles and venules, to mimic subsequent thrombotic events, and to definitively describe the potential for AnxA1 to be a treatment strategy in stroke. Figures 5G and 5H show that when AnxA1 is given after cerebral I/RI, it is able to significantly protect against subsequent thrombotic events by causing a significant increase in blood flow in venules by 45.51% and in arterioles by 58.09%, respectively.

### A Direct Effect of AnxA1 on Human Platelets: An Undiscovered Platelet Modulator

**Figure 6.**
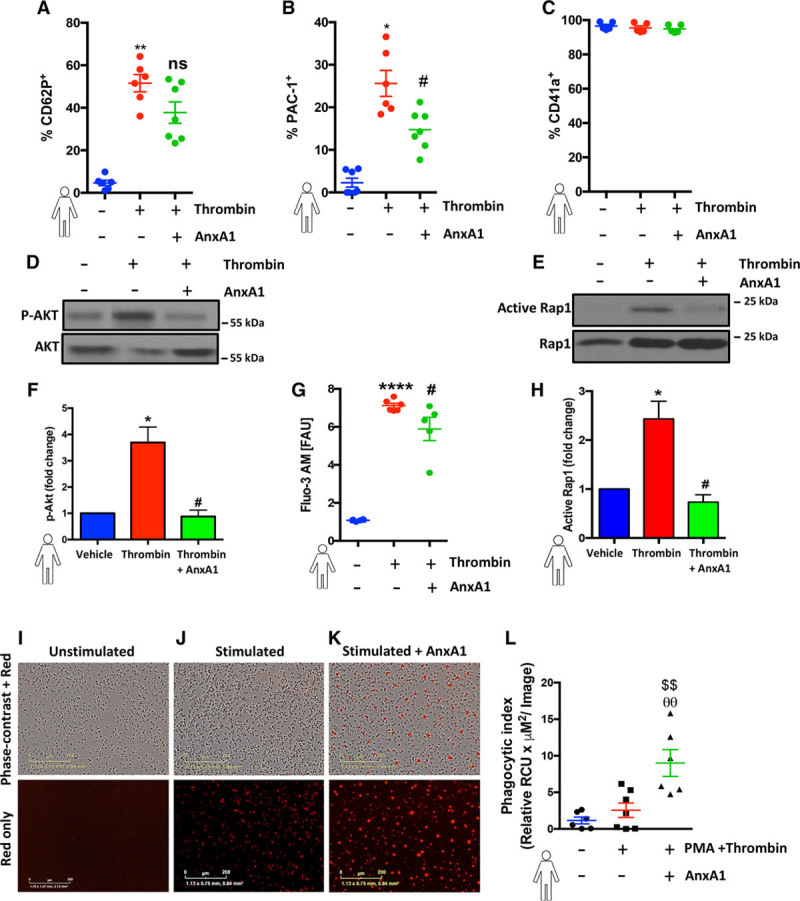
**Annexin A1 (AnxA1) decreases human platelet activation and promotes phagocytosis. A** through **C**, Human platelets (1×10^6^) were isolated, washed, preincubated with 100 ng of AnxA1 (30 min at 37°C), and stimulated with thrombin and using flow cytometry; levels of P-selectin expression (**A**), activated integrin α_IIb_β_3_ (PAC-1; **B**), and surface levels of α_IIb_β_3_ (CD41a; **C**) were quantified. **D** through **H**, Freshly isolated platelets (1×10^6^) were treated with AnxA1 at 100 ng and treated with vehicle (saline), thrombin (0.1 U), or thrombin and AnxA1 (100 ng) for 3 min, and cells were subsequently lysed and blotted for phospho-AKT (P-AKT) and total AKT (n=3 individual donors/group; **D** and **F**) and active Ras-associated protein 1 (Rap1) and Rap1 (n=5 individual donors/group; **E** and **H**) or changes cytosolic Ca^2+^ in fluo-3-acetoxymethyl ester (Fluo-3 AM)–labeled platelets was determined in the presence or absence of AnxA1 (**G**). **F** and **H**, Akt and Rap1 were quantified by ImageJ. The ratio of p-AKT/AKT and Active Rap1/Rap1 was calculated to get the fold change in reference to the untreated control. **G**, The graph shows the mean fluorescence intensity (fluorescent arbitrary units [FAU]) at baseline (no stimulation), in stimulated platelets (thrombin 0.1 U/1×10^6^ platelets), and in platelets incubated with AnxA1 (100 ng/1×10^6^ platelets) and stimulated with thrombin. **I** through **K**, Representative images of phagocytosis using pHrodo dye from 1 experiment at 1 h. The scale bars indicate 200 μm. **I**, Human platelets (1×10^6^) preincubated with 100 ng of AnxA1 and 2.5 μmol/L pHrodo Red AM intracellular pH indicator for 20 min, followed by opsonization with 1% human serum, with and without thrombin stimulation, were coincubated with neutrophils (with and without phorbol myristate acetate [PMA]) for 1 h at 37°C in a 96-well flat clear-bottomed black-walled plate, and fluorescence emission was measured in the IncuCyteZOOM (Essen BioScience) imaging platform. The data are means±SEM of 6 to 7 individual donors/group unless otherwise stated (2 outliers [defined as at least 2 standard deviations] were removed for **A**, and 1 outlier was removed for **B** and assessed by a Student *t* test (**A** and **B**), Kruskal–Wallis with Dunn’s test (**F** and **G**), or ANOVA with Bonferroni post hoc test (**C**, **H**, and **L**). *P<0.05, **P<0.01, and ****P<0.0001 vs unstimulated platelets. #*P*<0.05, ##*P*<0.01 vs thrombin-stimulated platelets. θθ*P*<0.01 vs unstimulated neutrophils and platelets. ^$$^*P*<0.01 vs PMA-stimulated neutrophils and thrombin-stimulated platelets. ns indicates not significant.

Finally, to further explore the translational relevance of the in vivo findings, we ascertained the direct effect of AnxA1 on human isolated platelets. Figure 6A and 6B shows that thrombin enhanced the surface levels of P-selectin and active α_IIb_β_3_ (as assessed by binding of FITC–PAC-1, a monoclonal antibody specific for neoepitopes exposed on the activated form of α_IIb_β_3_)^34^ on platelets, whereas direct treatment with AnxA1 inhibited only active integrin α_IIb_β_3_ levels (*P*<0.05), with no statistically significant effect on P-selectin levels. Interestingly, the effect of AnxA1 to reduce surface levels of active α_IIb_β_3_ was due to a decreased affinity of α_IIb_β_3_ for PAC1 rather than decreased surface expression of α_IIb_β, as shown using the α_IIb_β_3_ (CD41a) antibody (Figure 6C). In addition, we found that AnxA1 was able to inhibit thrombin-induced activation of classic inside-out signaling events such as Akt activation (Figure 6D and 6F), intracellular Ca^2+^ release (Figure 6G), and Rap1 activation (Figure 6E and 6H). These data suggest that AnxA1 may have a direct effect on integrin suppression and in so doing, reduces the propensity for platelets to aggregate and cause thrombosis.

It has previously been shown that P-selectin activates intracellular signaling pathways to initiate phagocytosis,^33^ and PS exposure on the plasma membrane is a property of activated platelets and cells undergoing apoptosis. Figure 6I through 6L demonstrates that AnxA1 is able to promote the phagocytosis of platelets by neutrophils. These data provide further evidence that not only is AnxA1 able to reduce the prothrombotic ability of platelets in vivo, but its effects are translated in the human setting and could provide a therapeutic mechanism by which AnxA1 is able to reduce thrombus formation.

## Discussion

**Figure 7.**
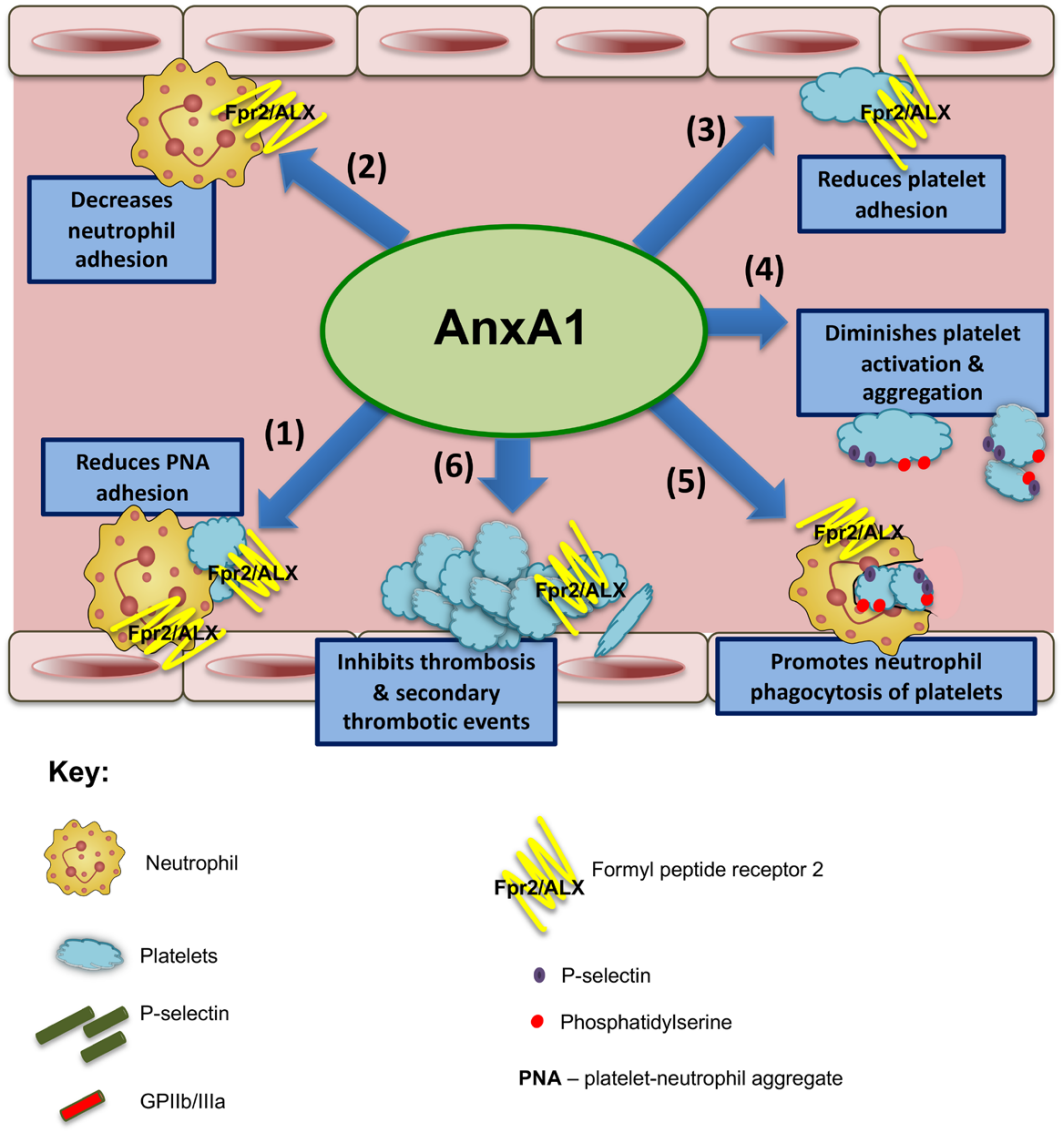
**Annexin A1 (AnxA1) mitigates thromboinflammatory responses and promotes resolution during ischemia reperfusion injury**.Schematic overview shows the effects of AnxA1 on both thrombotic and inflammatory events as follows. Step 1 reduces platelet–neutrophil aggregate (PNA) adhesion on endothelial cells in the cerebral microvasculature; steps 2 and 3 decrease neutrophil (step 2) and platelet adhesion (step 3); step 4 diminishes platelet activation and aggregate velocity, promoting a phosphatidylserine and P-selectin–dependent clearance program; step 5 promotes neutrophil phagocytosis of platelets for resolution; and step 6 protects against enhanced intravascular thrombus formation and secondary thrombotic events in the cerebral microvasculature. ALX indicates the lipoxin A4 receptor; and Fpr2, formyl peptide receptor 2.

**Figure 8.**
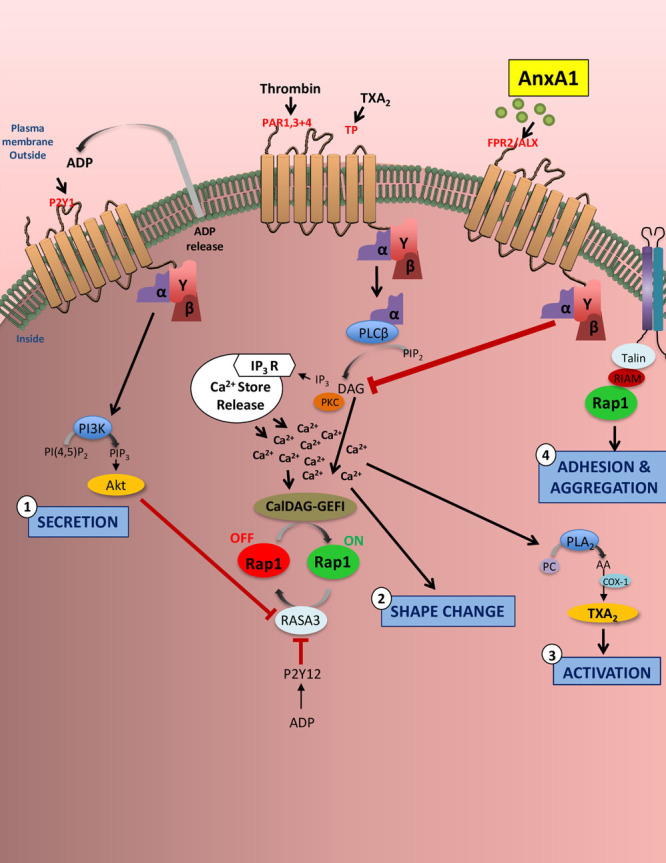
**Annexin A1 (AnxA1) reduces platelet activation and integrin activity.** In thromboinflammation, platelets are stimulated via G-protein–coupled receptors (eg, protease-activated receptors 1, 3, and 4; human thromboxane A2 receptor [TPs]; P2Y12; P2Y1; and P2X1) and immunoreceptor tyrosine-based activation motif-coupled receptors with agonists such as thrombin, thromboxane A2 (TXA_2_), and ADP. This stimulation results in downstream signaling events that increase cytosolic Ca^2+^ levels leading to platelet secretion (step 2), shape change (step 2), activation (step 3), and adhesion and aggregation (step 4). The small GTPase Ras-related protein 1 (RAP1) regulates multiple functional responses in platelets, in particular integrin activation. As with many platelet components, Rap1 regulators CalDAG–GEFI (calcium- and DAG-regulated guanine exchange factor-1) and RASA3 are affected by small changes in the cytosolic Ca^2+^ concentration, leading to rapid α_IIb_β_3_ integrin activation. To sustain α_IIb_β_3_ activation, ADP (which is rapidly released and secreted from storage granules on increased Ca^2+^ concentrations) must engage P2Y12 receptors and phosphatidylinositol 3-kinase (PI3K) signaling, which reduces RASA3 activity. AnxA1 acts via platelet formyl peptide receptor 2 (also known as the lipoxin A4 receptor; FPR2/ALX) to affect each of these 4 named platelet events, thereby inhibiting thromboinflammation. In particular, our data showed that AnxA1 reduced Akt expression, Ca^2+^ levels, Rap1 expression, and the affinity of α_IIb_β_3_ (the most abundant of the β_1_ integrins and β_3_ integrins expressed on the platelet surface) for PAC-1. The effects of AnxA1 on platelets point toward a novel and previously undiscovered role for AnxA1 to act as an antithrombotic agent by suppressing integrin activation and thereby reducing platelet activation, a prerequisite for platelet aggregation and thrombosis. AA indicates arachidonic acid; COX-1, cyclooxygenase 1; DAG, diacylglycerol; IP3R, receptor for inositol 1,4,5-trisphosphate; PC, phosphatidylcholine; PKC, protein kinase C; PLA2, phospholipase A2; and PLCβ, phospholipase Cβ.

We present herein several novel key findings. Firstly, the absence of AnxA1 heightens platelet adherence and platelet aggregate recruitment to the cerebral endothelium after I/RI. Exogenous administration of AnxA1 reduces both platelet activation and aggregation, promoting cerebral protection against thromboinflammation. Additionally, AnxA1 treatment prevented the development of subsequent thrombotic events after cerebral I/RI. Both murine and human stroke are associated with decreased circulating levels of AnxA1, which may help to explain the exacerbated thromboinflammatory response seen in stroke. Finally, we show that the AnxA1 is able to act on human platelets by inhibiting thrombin-induced activation of classic inside-out signaling (including Ca^2+^ influx and Akt activation) and selectively mediating cell surface determinants (eg, PS, P-selectin) to promote phagocytosis and trigger their clearance by neutrophils, thus initiating active resolution of thromboinflammation. Altogether, these novel findings for the first time demonstrate that the proresolving ligand AnxA1 is able to exert its protection through platelets via Fpr2/ALX by orchestrating the delicate balance of platelet function from a propathogenic to a regulatory role in cerebral I/RI (and potentially I/RI in other vascular beds). Thus, AnxA1 could be used as a viable treatment therapy in stroke to protect against I/RI by reducing the associated thromboinflammation (Figures 7 and 8).

There is an urgent medical need to decipher mechanisms underlying thromboinflammatory events to develop new therapeutic opportunities.^35,36^ A therapeutic drug-discovery program we are actively pursuing builds on the concept of resolution of thromboinflammation, with AnxA1 being a compound of significance.^7,9,37,38^ Although recent interest has been dedicated to effects of AnxA1 with respect to leukocytes (neutrophils, macrophages) in inflammation resolution, the role that platelets (which express Fpr2/ALX [Figure VI in the online-only Data Supplement], the key receptor involved in the resolution process), platelet aggregates, and microvascular occlusion play in both thrombosis and inflammation strongly suggests that these cells could be an additional target for AnxA1 and one that has not been previously studied. Here, we questioned whether we could exploit the biological properties of AnxA1 and its receptor FPR2/ALX to instigate possible novel drug-discovery programs for anti-inflammatory, proresolving strategies suitable to treat cardiovascular diseases especially acute ischemic stroke.^37,39^ Hence, we began here by assessing whether AnxA1 played functional roles in cerebral I/RI.

We demonstrated herein that a genetic *AnxA1* deletion is not associated with spontaneous thrombosis or bleeding but leads to exacerbated cerebral inflammatory responses after tMCAo/R. Specifically, these effects were detected by increased adherent platelets and heterotypic platelet aggregates in the pial circuit, which were coupled with increased neuronal and neurovascular damage, eg, increased infarct volume and a disrupted BBB. Moreover, the BBB of AnxA1^−/−^ mice was compromised to a greater degree in the ipsilateral side compared with control mice, suggesting a lack of AnxA1 either directly or indirectly promotes BBB permeability postischemic injury. Furthermore, the BBB was also more permeable in the contralateral side (ie, noninfarcted side) of AnxA1^−/−^ mice versus WT mice, an effect previously unknown. These data fit with other recent findings demonstrating that AnxA1^−/−^ mice have increased BBB permeability at resting state^40^ and that AnxA1 is essential for maintaining tight junctions in the BBB and repairing the permeabilizing effect of systemic lipopolysaccharide on the BBB.^40,41^ Additionally, indirect effects leading to BBB disruption and damaged microvascular endothelium^9,35,42^ could be increased IL-1β, TNFα, and IL-6 cytokine levels (as seen in AnxA1^−/−^ mice), which can induce vascular permeability and adhesion molecule expression.

Multiple cell types are involved in the pathophysiology of I/RI, including resident cells (such as vascular smooth muscle cells, astrocytes, microglial cells, neurons, etc.) and blood-borne immune cells (such as platelets, neutrophils, monocytes, etc.). We have previously hypothesized that a second potential target for the effects of AnxA1 might be microglial cells. When peptide AnxA1_Ac2-26_ was administered to minocycline-treated mice, its observed cerebroprotective effects on leukocyte adhesion to pia mater venules were attenuated in 2 different models of inflammation: endotoxin (lipopolysaccharide)^38^ and stroke (F.N.E. Gavins et al, unpublished data, 2018). Other groups have focused on neurons, although controversy has previously surrounded the localization of AnxA1 within these brain resident cells, possibly because of differences in antibody specificities, post-translational modification of AnxA1 in neurons to mask antigenicity, differences in tissue processing, or all of the above.^43^ Some studies using the AnxA1^−/−^ mouse (in which the gene for β-galactosidase, *LacZ*, has been inserted under the control of the AnxA1 promoter) have already been performed showing β-galactosidase staining in ependymal cells lining the ventricles, in intracerebral blood vessels, and in a highly restricted set of neurone-like cells in the Substantia nigra pars compacta and the pyramidal layer of the hippocampus.^44^ Since these findings were shown, other groups have shown AnxA1 to be involved in neuronal apoptosis via its phosphorylation^45^ or an interaction with p53.^46^

Interestingly, human and murine stroke is associated with decreased circulating levels of AnxA1. Given the nature of this antithromboinflammatory and proresolving nature of this protein, these decreased levels may help to explain the propagation of the inflammatory response in stroke and concurs with reduced levels found in other chronic inflammatory conditions, eg, sickle cell disease, Crohn’s disease, obesity, and sepsis.^47–50^

AnxA1 administration reduced both individual platelet and neutrophil adhesion in tMCAo/R mice. However, because cerebral-endothelial PNA adherence is a key effector of microvascular occlusion, we determined whether AnxA1 treatment would have any affect. Indeed, AnxA1 blocked cerebral endothelial recruitment of PNAs post-tMCAo/R, an effect mediated through Fpr2/ALX. These findings are important because both PNA formation and platelet activation are increased in ischemic stroke and are involved at several levels in the pathogenesis and severity of stroke.^51^ Although PNAs have been widely used as a surrogate marker for systemic inflammatory responses,^52^ the pathophysiological importance of these aggregates is unclear.^53^ The fact that AnxA1 reduced endothelial PNA formation in the brain further supports the concept of this protein acting as a key resolving mediator of thromboinflammation after cerebral I/RI.

Although administration of AnxA1 prevented cerebral PNAs adhesion, it did not stop aggregates forming in the circulation. It is tempting to speculate that the differences observed with AnxA1 administration on local and systemic PNA formation might be due to clearance and phagocytosis to promote resolution of thrombosis and inflammation. Platelet clearance is comprised of 2 specific events: adhesion to neutrophils (via P-selectin and PSGL-1) and internalization into the white blood cell.^54–57^ The extent of platelet clearance can correlate with platelet activation, often monitored through elevated P-selectin expression.^55^ We found here that AnxA1 limited the prothrombotic and proinflammatory impact of both platelet–platelet and platelet–neutrophil aggregates. Also, AnxA1 did not change P-selectin expression on platelets, which may be because recognition of P-selectin activates intracellular signaling pathways to initiate phagocytosis.^55^ Evidence suggests that to promote phagocytosis, AnxA1 acts as a ligand for 2 specific sites, binding through its core domain to PS on the surface of apoptotic neutrophils and to Fpr2/ALX via its N-terminal sequence.^58^ Maderna *et al*.^58^ have shown that Fpr2/ALX expression and phosphokinase C–dependent internalization of Fpr2/ALX is required for phagocytosis and clearance of activated neutrophils by bone marrow–derived macrophages. In addition, Ca^2+^ mobilization (activation of phospholipase C) promotes platelet apoptosis and elimination of platelets from circulation, and AnxA1 and its bioactive peptides are able to mobilize intracellular Ca^2+^ in neutrophils.^59^ As such, engagement of Fpr2/ALX by AnxA1 on platelets may therefore cause Ca^2+^ mobilization, promoting platelet apoptosis.^60^

Having studied the effects of AnxA1 on PNAs and neutrophil involvement in PNAs, we next focused on the platelet, especially because no studies had previously defined the bio-actions of AnxA1 on platelets. Platelets not only participate in hemostasis and thrombosis but can also modulate acute and chronic inflammatory responses. However, the mechanisms underlying the prothrombotic phenotype generated by cerebral I/RI remain poorly understood. Here, we found that platelet adhesion and platelet heterotypic aggregates were upregulated in stroke (and further increased in AnxA1^−/−^ mice, although no differences were found in platelet counts) and were reduced in the brain of tMCAo/R mice after administration of AnxA1. It is known that when platelets are activated, they release thromboxane A_2_ and externalize PS, which, in cooperation with other clotting factors (Va and Xa, and Ca^2+^-forming prothrombinase complex),^61,62^ initiate the intrinsic coagulation pathway forming thrombin, platelet–platelet, and platelet–neutrophil aggregates and intensifying platelet accumulation to develop stable thrombi.

Previously, no direct information was available on Fpr2/ALX-mediated intracellular effects regulating platelet aggregation. Because Fpr2/ALX is a G-protein–coupled receptor, it is tempting to speculate that the engagement of Fpr2/ALX with AnxA1 may trigger activation of adenylate cyclase (as evidence by AnxA1 treatment decreasing thromboxane B_4_ plasma levels) and elevation of cAMP or/and cGMP levels in platelets leading to the decreased aggregation velocity, as observed in our study.^60,61,63^ We found that not only were plasma levels of thromboxane B_4_ decreased after AnxA1 treatment, but AnxA1 also reduced PS exposure on single platelets, as well as on their aggregates, along with reducing the velocity of aggregate formation. The results suggest that after cerebral ischemia, AnxA1 is a nonredundant factor that promotes an anti-inflammatory/antithrombotic and proresolving environment.

Integrins are critical in thrombosis and hemostasis, supporting platelet–matrix (platelet adhesion) and platelet–platelet interactions (platelet aggregation).^64,65^ Currently available antithrombotic agents target the interaction of platelet integrin α_IIb_β_3_ with fibrinogen during platelet aggregation but are associated with life-threatening adverse effects of bleeding.^2,3,66^ Integrin α_IIb_β_3_ (the most abundant integrin expressed on the platelet surface)^67^ plays a central role in thrombosis and hemostasis, because of its ability to mediate platelet adhesion and aggregation. In resting states, this major membrane protein expressed on the platelet cell surface membrane and intracellular compartments.^68^ However, on activation (eg, by thrombin), inside-out signaling is initiated and induces a cation-dependent transformation of receptors from a low-affinity to a high-affinity state, which is dependent on elevated intracellular Ca^2+^.^67–69^ Conversely, outside-in signaling mediates platelet spreading and also greatly amplifies platelet thrombi.^70^ Because the activation of integrin α_IIb_β_3_ is the final common pathway of platelet activation, the assessment of its activation is critical for accurate studies on platelet function.^68,71^ We found here that AnxA1 was able to inhibit thrombin-stimulated increases in active α_IIb_β_3_ integrins on human platelets (as assessed by the ligand-mimetic monoclonal antibody PAC-1, which detects specifically high-affinity conformation of integrin α_IIb_β_3_.^68^ In addition, the use of CD41a and PAC-1 in flow cytometry allowed us to discriminate between the resting and the activated form of the receptor because platelet activation is a prerequisite for platelet binding.^68^

Activation of the small GTPase Rap1 promotes its interaction with talin1, inducing talin1’s interaction with a membrane-proximal region of the β-integrin tail that drives a conformational change in the integrin extracellular domain enhancing its affinity for ligand.^67^ Thrombin enhances α_IIb_β_3_ affinity through the Ca^2+^-dependent activation of the guanine nucleotide exchange factor CalDAG, which facilitates Rap1 activation. However, sustained Rap1 signaling requires Ca^2+^-dependent ADP release and P2Y12-dependent phosphatidylinositol 3-kinase signaling, which inactivates the Rap1 inhibitor RASA3. We found that AnxA1 was able to inhibit thrombin-induced activation of Ca^2+^ influx, ADP release, and activation of the phosphatidylinositol 3-kinase effector Akt (Figure 6D through 6G). Consistent with this, our data show that AnxA1 is able to decrease the Rap1 activation in thrombin-stimulated platelets. In addition to inside-out integrin activation, integrin engagement also leads to outside-in integrin signaling, resulting in changes in intracellular signaling pathways (eg, activation of Akt and increased intracellular Ca^2+^ release). This increase in intracellular Ca^2+^ along with PS exposure are associated with an enhanced procoagulant activity, thereby driving thrombogenesis and microvascular occlusion. The regulation of integrin signaling is a critical aspect in multiple steps of platelet function and thrombus formation.^71,72^ Collectively, these data point toward a novel and previously undiscovered role for AnxA1 to act as an antithrombotic agent by suppressing integrin activation and thereby reducing platelet activation, a prerequisite for platelet aggregation and thrombosis. Additionally, these data could pave the way to novel and more personalized approaches to antiplatelet therapy.

Interestingly, the prothrombotic ability of platelets also directly relates to the degree of PS exposure, such that a reduction of PS exposure on the surface of platelets links to reduction of thrombus formation.^61,62^ Here, we found a previously unknown role of AnxA1 to increase blood flow cessation time because we were able to demonstrate, for the first-time, the dynamics of AnxA1 on thrombosis induced using the light/dye method.^25^ AnxA1 administration was equally effective in both arterioles and venules, despite the known physical and rheological differences in each of these vessels types.^1,73^ As such, our findings emphasize the possible therapeutic potential of AnxA1 as a preventative measure, as well as a novel treatment strategy for stroke.

Finally, having demonstrated the protective effect elicited by AnxA1 postcerebral I/RI coupled with the protection afforded by the protein against cerebral thrombosis (a prerequisite for cerebral ischemia), we determined whether we could further exploit the effects of AnxA1. As such, we treated mice with AnxA1 before stroke and then performed light/dye thrombosis. Our novel findings show that AnxA1 was able to increase blood flow cessation after a subsequent thrombotic event postcerebral ischemia, thereby showing the diverse nature of AnxA1.

In summary, our results demonstrate that the lack of AnxA1 leads to aberrant platelet recruitment and aggregation in the brain after ischemic stroke and that exogenous administration of AnxA1 is a powerful proresolving mediator of thromboinflammation. We report previously unknown effects of AnxA1 on platelets, including a possible endogenous circuit in which AnxA1 modulates the expression of platelet moieties (eg, PS and P-selectin) and as such can promote phagocytosis and trigger platelet clearance by neutrophils. Furthermore, we provide novel evidence that AnxA1 is not only able to reduce the effects of cerebral I/RI (including reducing the effect of subsequent thrombi forming after cerebral ischemia), but it is able to reduce cerebral thrombosis, a prerequisite for stroke. These novel physiopharmacological properties for the AnxA1 and FPR2/ALX pathway increase our understanding of the cellular intravascular events typical of stroke and open new avenues for an attractive therapeutic treatment strategy not only for patients with stroke but also for patients susceptible to stroke.

## Acknowledgments

We thank Dr Gregory Del Zoppo (University of Washington, Seattle, WA) for his generous help with the manuscript and Seth Fruge (LSUHSC-S) for help with clinical data collection.

## Sources of Funding

This work was supported by National Institutes of Health grants HL134959-01A1 (to Dr Gavins); HL098435, HL133497, HL141155, and GM121307 (to Dr Orr); GM121307 (to Dr Stokes); R01 HL142604-01 (to Dr Pawlinski); and RO1CA192111 (to Dr Han). This work is also supported by American Heart Association grant 19PRE34380751 (to Dr Al-Yafeai) and Wellcome Trust grant 086867/Z/08/Z (to Dr Perretti).

## Disclosures

None.

## Supplementary Material
