# Supplementary material for: Novel Role for the AnxA1-Fpr2/ALX Signaling Axis as a Key Regulator of Platelet Function to Promote Resolution of Inflammation
Source: Circulation. 2019 Jun 3;140(4):319–35. doi: 10.1161/CIRCULATIONAHA.118.039345 (PMC6687438; doi:10.1161/CIRCULATIONAHA.118.039345)
Supplement: Supplementary file 1 [file cir-140-319-s001.pdf]

SUPPLEMENTAL MATERIAL

Supplementary Methods

The data that support the findings of this study are available from the corresponding author upon reasonable request.

Animals

All studies were performed on adult male mice weighing 25-30g. Wild-type (WT) C57BL/6 mice were purchased from either Jackson Laboratory (Bar Harbor, ME, USA) or Charles River Limited (Margate, Kent, UK). AnxA1$^{-/-}$ mice were backcrossed for over twelve generations on a C57BL/6 background[1] and bred on site. The AnxA1$^{-/-}$ mice showed no obvious phenotype and were fertile. Mice were maintained on a 12 hours (h) light-dark cycle during which room temperature was maintained at 21–23°C, and had access to a standard chow pellet diet and tap water *ad libitum*. All animal experiments complied with ARRIVE (Animal Research: Reporting In Vivo Experiments) guidelines and followed the European Union Directive (2010/63/EU) or approved by the Louisiana State University Health Sciences Center-Shreveport (LSUHSC-S) Institutional Animal Care and Use Committee (IACUC) and were in accordance with the guidelines of the American Physiological Society.

Human

The study was approved by the institutional review board of the LSUHSC-S (STUDY00000572 and STUDY00000261) and conducted in accordance with the Declaration of Helsinki. The consent form was discussed and after written permission obtained, blood was taken from control volunteers (38-73 years old, five males, 3 females). Stroke patients (45-89 years old, five males, six females) were recruited upon admission to the hospital for mechanical thrombectomy at the LSUHSC-S

and consent was obtained during their post-operative stay at the hospital. Blood was collected at the same time as mechanical thrombectomy. Additional clinical data was also collected from the patient's medical records, including demographic features, cerebrovascular risk factors, probable stroke etiology (cardiac, carotid or intracranial), pre-operative National Institutes of Health Stroke Scale (NIHSS) scores, radiological data (artery involvement) and hematological parameters. Patients with acute infection or other chronic blood borne diseases (HIV, Hepatitis B/C) were excluded from the study.

Receptor agonists and drug treatment

Vehicle (saline), AnxA1 (Cambridge Research Biochemicals, Cleveland, UK) 3.3 mg/kg,[1] WRW4 (Tocris, Bristol, UK) 1.8 mg/kg,[2] were administered intravenously (i.v.) at the start of cerebral reperfusion. Doses chosen were based on previously published data.[1,2] All studies were performed blinded and randomized, with a key system to identify which animal/sample had undergone which treatment. Furthermore, compounds administered were made by laboratory personnel other than the one performing the experiment.

AnxA1 quantification plasma

Human or Murine AnxA1 ELISA kits (MyBioSource, San Diego, CA, USA) were used to quantify the plasma levels of AnxA1. Plasma preparation and the ELISA assay were done according to manufactures' instructions. Results were reported as pg/ml or ng/ml of AnxA1 concentration in plasma from control volunteers and stroke patients or mice with and without stroke (transient middle cerebral artery occlusion for 60 min followed by 24 h reperfusion).

Transient focal middle cerebral artery occlusion with reperfusion (tMCAo/R)

As a cerebral ischemia reperfusion model, transient middle cerebral artery occlusion with reperfusion (tMCAo/R) was performed as previously reported.[3] Briefly, mice were anaesthetized with i.p. injection of ketamine (150 mg/kg) and xylazine (7.5 mg/kg) and MCA was occluded for 60 minutes (min) using a 6-nylon intraluminal filament (Doccol Corporation, Massachusetts, USA), followed by 4 h and 24 h of reperfusion. Sham-operated mice were subject to anesthesia and other surgical procedures without MCA occlusion.

Platelet and leukocyte labeling

Blood from donor mice was withdrawn in syringes prefilled with 85 mM sodium citrate, 62.2 mM citric acid, and 110 mM glucose (ACD buffer, Sigma-Aldrich, St Louis, MO). Platelet-rich plasma was obtained by centrifugation (118 × *g*, 8 min) before platelet isolation by centrifugation at 735 *g* for 10 min. Platelets were gently resuspended, counted, and labeled by using carboxyfluorescein succinimidyl ester (CFSE, 90 μM, 10 min, Sigma-Aldrich). After confirming absence of aggregates by light microscopy, 100×10^6 platelets in 120 μL saline were injected through the jugular vein of a recipient mouse over 5-min infusion as previously described.[3] This was followed by the continuous infusion of 0.02 % rhodamine 6G, which fluorescently labeled circulating leukocytes.

Neutrophil depletion

In some experiments, neutropenia was induced using mouse anti-neutrophil serum (ANS; 1A8; BioXCell, West Lebanon, NH, USA). ANS was administered (150 μg/mouse) i.p. 24 h before the experiment.[4] A reduction in circulating neutrophils was verified by counting neutrophils in the

peripheral blood before ANS injection and immediately before the experiment. ANS did not alter the number of circulating lymphocytes, monocytes, or platelets.

Neutrophil isolation and adoptive transfer

Mouse bone marrow cells were harvested by flushing marrow from femurs and tibias with RPMI-1640 medium. Neutrophils were isolated from bone marrow cells by density centrifugation with Histopaque-1077 and Histopaque-1119. The purity of isolated neutrophils was routinely > 95% as assessed by light microscopic analysis of the cells stained with Diff-Quick (Wako Pure Chemical Industries, Osaka, Japan), and > 98% viable as assessed by a trypan blue exclusion test. The neutrophils were labelled with CellTracker dye (ThermoFisher, Waltham, MA) at a final concentration of 5 µM,[5] and then treated with WRW4 (10µM. Tocris)[6] for 10 min prior to injection (5 x 10^6) through the jugular vein of a recipient neutropenic MCAo/R mouse. Mice were then treated with AnxA1 (1 µg/mouse) 30 min prior to intravital microscopy (IVM).

Cerebral intravital fluorescence microscopy (IVM)

IVM was performed as previously described.[2] Briefly, mice were re-anaesthetized with i.p. injection of ketamine (150 mg/kg) and xylazine (7.5 mg/kg). The jugular vein was cannulated for i.v. administration of rhodamine 6G and *ex vivo* labeled platelets. The carotid artery was cannulated with PE-10 tubing and mean arterial blood pressure (MABP) and heart rate (HR) were continuously recorded by a MacLab system (AD Instruments, Chalgrove, UK). The head of each mouse was then fixed in a plastic frame in sphinx position. The left parietal bone was exposed by a midline skin incision, followed by a craniectomy (diameter: 2.5 mm) with a drill at 1 mm posterior from the bregma and 4 mm lateral from the midline. The dura mater was not cut because

the fluorescent-labeled leukocytes were readily visualized. A 12 mm glass coverslip was placed over the craniectomy and the space between the glass and dura mater was filled with artificial cerebrospinal fluid (aCSF; Na^+ 147.8 mEq/L, K^+ 3.0 mEq/L, Mg^{2+} 2.3 mEq/L, Ca^{2+} 2.3 mEq/L, Cl^- 135.2 mEq/L, HCO_3^- 19.6 mEq/L, lactate$^-$ 1.67 mEq/L, phosphate 1.1 mM, and glucose 3.9 mM; all Sigma-Aldrich). A Zeiss Axioskop microscope (Zeiss, New York, USA) with a mercury lamp was used to observe the pial venules in the cerebral cortex. A Hitachi charge-coupled device color camera (model KPC571; Tokyo, Japan) acquired images that were recorded for offline analysis. One to five randomly selected vessels, 30–70 μm in diameter and 100 μm long, were observed for each mouse after treatment. Adherent leukocytes and platelets were defined as cells remaining stationary within venules for ≥30 seconds (s) or ≥2 s respectively. Platelet-leukocyte aggregates (PLAs), were defined as platelets interacting directly with adherent leukocytes on the endothelium. These parameters were expressed as the number of cells per square millimeter of the vessel surface and calculated from diameter and length, assuming cylindrical shape. Estimates of pseudoshear rate were obtained using measurements of vessel diameter (D_v) and the maximal velocity of flowing red blood cells (V_{cell}), according to the following formulation: pseudo-shear rate = $(V_{cell}/1.6)/D_v \times 8$.[4]

Confocal Microscopy

To visualize platelet neutrophil aggregate (PNA) formation, mice were injected with specific antibodies to label: neutrophils (eFluor 488 (green)-labeled anti-Mouse Ly-6G, eBioscience, San Diego, CA. 2μg/mouse) and platelets (Dylight 649 (red)-labeled anti-mouse CD42, (Emfret Analytics, Eibelstadt, Germany. 1μg/mouse), as previously described.[3] Mice were placed on an Olympus BX51WI upright microscope (Olympus, Center Valley, PA) with a 20X (LUCPlanFLN)

objective and equipped with a 3i LaserStack laser launch (3i, Denver, CO), Yokogawa CSU-X1-A1N-E spinning disk confocal unit (Yokogawa Electric Corporation, Tokyo, Japan) and electron multiplier CCD camera (C9100-13, Hamamatsu, Bridgewater, NJ), in order to visualize PNAs. Slidebook software (3i) was used to drive the confocal system and capture images.[3]

Systemic PNA assessment

Murine blood obtained by tail-vein bleed before and after tMCAo was mixed with heparin (20U/mL), as described previously.[8] The samples were treated with rat anti-mouse CD16/CD32 antibody to reduce non-specific antibody staining. The samples were diluted with phosphate buffered saline and then stained, followed by lysis of the red blood cells. A four-color flow cytometry assay was used to separate the PNAs: Leukocytes were labeled with rat anti-mouse CD45.2-FITC, Gr-1-PE, and F4/80-eFluor450 and isotype controls (eBioscience, San Diego, CA) while platelets were labeled with CD41-APC. CD45.2 and CD41 double-positive events were identified as a percentage of 10,000 gated leukocyte events. The percentage of neutrophil forming aggregates was calculated from the number of platelet leukocyte aggregates/µl blood divided by the number of neutrophils/µl blood x 100.

Assessment of activated murine platelets by flow cytometry

0.9 mL of whole blood was collected via carotid artery into syringe containing 0.1mL ACD buffer, transferred to an eppendorf tube, and centrifuged at 1200 rpm for 8 min. The platelet-rich plasma layer was transferred to a new eppendorf tube and centrifuged at 1200 rpm for 3 min. The Platelet rich plasma (PRP) was transferred and centrifuged at 3000 rpm for 10 min. The supernatant was discarded and the platelet pellet was resuspended in Tyrodes buffer (with 1 mM Ca^{2+}) buffer along

with the appropriate antibody and allowed to incubate for 15 min at 37 ^{0}C.The reaction was stopped by the addition of 450 μL of 1% paraformaldehyde. Two-color staining of $\alpha_{IIb}\beta_3$ (Integrin $\alpha_{IIb}\beta_3$-PE (clone JON/A. BD Pharmingen, San Jose, CA, USA)) and P-selectin FITC (Emfret Analytics) were used to measure levels of activated $\alpha_{IIb}\beta_3$ and P-selectin expression on platelets stimulated with either with thrombin 0.1 U/1x10^6 platelets or ADP 1 μM using flow cytometry (FACS Calibur). AnxAV FITC (BD Pharmingen, San Jose, CA, USA) was also used to measure phosphatidylserine (PS) externalization. IgG isotype antibodies were used as controls. Blood samples were stimulated and stained with antibody for 15 min at RT. Platelets and platelet aggregates were identified by their light scattering using an LSRII flow cytometer and Diva8 software by assessing at least 20,000 events that were collected per sample.

Assessment of activated human platelets by flow cytometry

5 ml of whole blood obtained from healthy volunteers was collected into a 10 ml syringe containing 1mL of ACD buffer. Blood was transferred to a 15 ml conical tube and centrifuged at 300 g for 5 min. The PRP layer was transferred to a new eppendorf tube and centrifuged at 3000 rpm for 10 min. The supernatant was discarded and the platelet pellet was resuspended in Tyrode`s buffer (with 1 mM Ca^{2+}). The platelets were treated with Fc block for a 15 min incubation period at room temperature. The platelets were then placed in the flow tubes containing Tyrodes buffer along with the appropriate antibody to measure platelet activation using an antibody targeting the activated form of $\alpha_{IIb}\beta_3$ (CD41/CD61-AlexaFluor 647 [clone PAC-1], Biolegend, San Diego, CA, USA), platelet GPIIb/IIIa complex (CD41a-eFluor 450 [eBioMWReg30], Thermofisher) and P-selectin (CD62P-FITC, Biolegend) and incubated for 15 min. Platelets were treated with AnxA1 (100 ng per 1x10^6 platelets), incubated for 30 min and stimulated with thrombin 0.01 U for 2 min. The

reaction was stopped by the addition of 450 μL of 1% paraformaldehyde. Platelets were identified by their light scattering using an LSRII flow cytometer and Diva8 software by assessing at least 20,000 events that were collected per sample.

Platelet aggregation assay

Arterial blood was freshly collected from the carotid artery of tMCAo/R-WT mice treated with either vehicle (saline) or AnxA1 into 1 ml syringe loaded with anticoagulant citrate dextrose (ACD) with 0.2 u/ml of apyrase (final concentration) (apyrase, grade VII, Sigma-Aldrich) in a 1/7 ratio. PRP was obtained immediately by centrifugation at 140 g for 8 min, and then top layer of PRP with RBC was harvested into new Eppendorf tube and centrifuged again at 60 g for 4 min to remove RBC contamination (Thermo Scientific Legend Micro 17 centrifuge). Platelets 8-10 x 10^6/ml were used to monitor platelet aggregation velocity after agonist exposure using a laser-particle analyzer (LasCa-1C, Lumex Ltd., St. Petersburg, Russia) by low angle light scattering method as previously described[9] in 6 ml of platelet media (140 mM NaCl. 10 mM HEPES, 10 mM NaHCO3, 2 mM KCl, 1 mM MgCl2, 2 mM CaCl2, 5.5 mM D-glucose, pH 7.4). Platelets were stimulated with either 0.1 u/ml of thrombin (Sigma-Aldrich a) or ADP 1 μM (Sigma-Aldrich) and normalized velocity of aggregation was calculated using original software LasCa_32.

Thrombosis

10mg/kg of 5% FITC dextran (150,000 mol wt, Sigma-Aldrich a) was injected intravenously, allowed to circulate for 10 min before photoactivation. Photoactivation was initiated (excitation, 495 nm; emission, 519 nm) by exposing 100μm of vessel length to epi-illumination, with a 175-W xenon lamp (Lamda LS, Sutter) and a fluorescein filter cube (HQ-FITC, Chroma).[6] The

excitation power density was measured daily (ILT 1700 Radiometer, SED033 detector; International Light, MA) and maintained within 1% of 0.77 W/cm^2, as previously described.[6] Epi-illumination was applied continuously, and time of blood flow cessation (≥60s duration) was recorded in both venules and arterioles (30-70μm). Epi-illumination was discontinued once blood flow ceased in the vessel under study. Typically, 2–4 thrombi were induced in each mouse, and the results of each vessel type (venules, arterioles) were averaged.[6]

Rap1 activity

Rap1 activity assay was performed according to instructions from the supplier (Cell Signaling, Danvers, MA, USA). Briefly, platelets were isolated from control volunteers (1 x 10^7 platelets) and immediately treated with vehicle (saline), thrombin (0.1U) or thrombin and AnxA1 (100 ng for 30 min) for 3 min, then spun for 5 min and washed with cold PBS. Next, platelets were lyzed in lysis buffer (provided by Cell Signaling) and 1 mM phenylmethylsulfonyl fluoride (PMSF), then centrifuged for 15 min. Glutathione beads and GST-Ral GDS-RBD were added to each sample and incubated for 1 hour at 4°C. Finally, samples were washed and the eluents were prepared for western blotting to detect Active Rap1 using the using rabbit-anti-Rap1 antibody (Cell Signaling). Data were quantified as Rap1-GTP/Rap1 using ImageJ. The ratio of Active Rap1/Rap1 was calculated to get the fold change in reference to the untreated control.

Western blotting

Freshly prepared platelets (1x10^7 cells) were incubated with AnxA1 (100ng/1x10^6) for 15 min followed by thrombin stimulation (0.1 U) for 3 min. Platelet pellets were prepared for western blotting and rabbit anti-phospho-Akt1 (S473. Cell Signaling Technology, Boston, MA, USA),

rabbit anti-β-Tubulin (1:1000. Cell Signaling Technology), goat anti-Akt1 (1:500. Santa Cruz Biotechnology, Dallas, Tx, USA) or rabbit anti-FPR2 (1:1000. Abcam, Cambridge, MA, USA) were used as primary antibodies. Secondary antibodies used were HRP-conjugated goat anti-rabbit IgG or rabbit anti-goat IgG. Bands were visualized by incubating antibody-labeled membranes with chemiluminescence reagent (PerkinElmer Life Sciences, Rigaweg, Groningen, Netherlands) and exposed for autoradiography. Bands were quantified using ImageJ analysis. Analysis of total Akt and p-Akt1 was quantified as a ratio of the protein of interest to a loading control to get the fold change in reference to the untreated control.

Measurement of intracellular Ca^{2+} levels in Fluo-3-acetoxymethyl ester (Fluo-3 AM) loaded platelets

5 ml of whole blood obtained from healthy volunteers was collected into a 10 ml syringe containing 1 ml of ACD buffer and PRP was obtained by centrifugation 300 x g for 5 min. Next, a platelet pellet was obtained by centrifugation at 3000 rpm for 10 min and resuspended with PBS. Washed platelets (1 x 10^6) were stained with Fluo-3 AM (5μM) for 30 min at 37^0C as previously described[10] and some samples were incubated with vehicle (saline) or AnxA1 (100 ng) prior to thrombin stimulation. Fluorescent intensity of Fluo-3 AM loaded platelets was immediately recorded using BD LSR II (as previously described)[10] to determine a baseline of Fluo-3 AM loaded platelets and immediately following stimulation with human alpha thrombin (0.1 U/1x10^6 platelets). The mean fluorescent intensities of samples was analyzed using BD FACSDiva Software v 8.0.1) and data plotted as fluorescent arbitrary units (FAU).

Phagocytosis assay

Fresh platelets (30 x 10^6) isolated in PBS with no calcium were incubated with 2.5 µM pHrodo™ Red AM Intracellular pH Indicator (Molecular Probes, Eugene, OR, USA). AnxA1 100 ng/1 x 10^6 platelets was added in the treatment groups and the cells were incubated for 20 min at room temperature in the dark. Samples were opsonized with 1% of human serum and incubated for additional 10 min. Samples were centrifuged at 3000 rpm for 7 min. The resultant pellet was resuspended in PBS with calcium. The platelets were counted and then plated (1 x 10^6) into 96-well flat clear bottom black walled polystyrene tissue-culture (BD BioSciences, Franklin Lakes, NJ, USA). Neutrophils (50,000/well) were added to platelets and co-incubated for 10 min before adding buffer (DMEM with 3% fetal calf serum (FCS)) and stimulating platelets with thrombin (0.5 U) and neutrophils with Phorbol myristate acetate (PMA. Sigma-Aldrich. 50 nM) in the corresponding wells. The samples were centrifuged at 1000 rpm for 5 min to allow the cells to settle. The plates were transferred into the IncuCyte ZOOM® (Essen Biosceince, Inc, Ann Arbor, Michigan, USA) platform which was housed inside a cell incubator at 37°C/5% CO_2, until the end of the assay. Phase-contrast and fluorescent images were acquired (four images per well) using a 10X objective lens every 30 minutes for a total of 2h. IncuCyte™ Imaging Software was used to calculate the "Total Red Object Integrated Intensity (RCU x μm^2/image)". This value was normalized to the "Total Red Object Integrated Intensity (RCU x μm^2/image)" of the positive control (10^6 platelets loaded in acidic media [pH3]), to generate the "Phagocytic index" for the individual conditions. Red channel acquisition time was 800 ms.

Cytokines in Plasma and Brain Tissue

24 h after reperfusion, blood was collected into heparin-coated syringe, and plasma was obtained. After a 5 min transcardial perfusion with PBS, brain hemispheres were homogenized, sonicated,

and centrifuged in PBS containing protease inhibitors (Bio-Rad, California, USA). Levels of the pro- and anti-inflammatory cytokines: interleukin-1β (IL-1β), tumor necrosis factor alpha (TNFα), monocyte chemoattractant protein-1 (MCP-1 or CCL2), IL-10 and IL-6, were measured using standard ELISAs (Quantikine™ immunoassay kits, R&D Systems, Abingdon, UK). ATL and thromboxane B_2 (TXB_2) concentrations in plasma and brain homogenates were determined using ELISA kits from Neogen and GE Healthcare, respectively. We followed the manufacturer's specifications for lipid extraction. Cytokine concentrations were expressed as either pg/ml (plasma) or pg/g brain weight (brain).

Infarct volume (IV)

After a 24 h reperfusion period, mice were euthanized with an overdose of anesthetic and brains were immediately removed and placed into 4°C phosphate-buffered saline (PBS, Sigma-Aldrich) for 15 min; 2 mm coronal sections were then cut with a tissue cutter. The brain sections were stained with 2 % 2,3,5-triphenyltetrazolium chloride (TTC) in phosphate buffer at 37°C for 15 min and fixed by immersion in 10% formaldehyde.[3] The stained sections were photographed and the digitized images of each brain section (and the infarcted area) were quantified using a computerized image analysis program (NIH 1.57 Image Software).

Neurological score

The functional consequences of cerebral I/RI were evaluated by using a five-point neurological deficit score (0, no deficit; 1, failure to extend right paw; 2, circling to the right; 3, falling to the right; and 4, unable to walk spontaneously)[3] and were assessed in a blinded fashion. A maximal score of 4 could be assigned to each experimental animal.

Blood–Brain Barrier (BBB) Permeability

BBB permeability was assessed using the Evans blue (EB) extravasation, as described previously.[3] Briefly, a 2 % solution of EB (Sigma-Aldrich) was injected (4 mL/kg) i.v. 24 h later, blood was obtained for plasma collection and brains were harvested after transcardial perfusion with PBS for 5 min. The cerebral hemispheres and the plasma were homogenized in 50 % trichloroacetic acid, sonicated, and then centrifuged. The supernatant was diluted with ethanol and the concentrations of Evans blue in brain tissue and plasma were measured using a fluorescence spectrophotometer (FLUOstar Optima, BMG LABTECH, Inc. Ortenberg, Germany). BBB permeability was normalized by dividing tissue EB concentration (µg/g brain weight) by the plasma concentration (µg/mL).

Thrombin-antithrombin (TAT) complex measurement

Plasma was collected from WT and $AnxA^{1-/-}$ mice and TAT was measured using standard TAT complex ELISAs (Assay Pro, St. Charles, Missouri, USA).[11]

D-dimer

Plasma was collected from WT and $AnxA1^{-/-}$ mice and D-dimer was measured using Asserachrom D-dimer kit (no. 00947. Diagnostica Stago, Parsippany, New Jersey, USA).

Statistical Analysis

Results from IVM experiments were confirmed to follow a normal distribution using Kolmogorov-Smirnov test of normality with Dallal-Wilkinson-Lillie for corrected *p* value. Data that passed the

normality assumption was analyzed using Student's *t*-test (two groups) or ANOVA with Bonferroni post-tests (more than two groups), which were performed using GraphPad Prism5 software. Data that failed the normality assumption were analyzed using the non-parametric Mann-Whitney U test (two groups) or Kruskal-Wallis with Dunn's test (more than two groups). Data are shown as mean values ± standard error of the mean (SEM), or median with interquartile range (neurological score only). Differences were considered statistically significant at a value of $p < 0.05$.

Supplemental Table 1. Hemodynamic parameters of WT and AnxA1$^{-/-}$ mice.

Genotype	Number	Body weight (g)	Treatment	Vessel diameter (µm)	pseudo-shear rate (per s)	Mean arterial blood pressure (mm Hg)
WT	6	29.4 ± 0.4	Sham	35.2 ± 4.5	585 ± 69	77.4 ± 5.2
	6	30.5± 0.5	I/R	40.1 ± 2.3	475 ± 76	80.3 ± 4.8
AnxA1$^{-/-}$	6	31.2 ± 0.4	Sham	40.2 ± 1.4	579 ± 89	78.4 ± 5.1
	6	29.8± 0.5	I/R	39.2 ± 2.1	468 ± 101	80.4 ± 3.9

WT = Wild-type mice. AnxA1$^{-/-}$ = Annexin A1 knockout mice. I/R = 60 min ischemia + 24 h reperfusion. g = grams. µm = micrometer. per s = per second mmHg = millimeter of mercury.

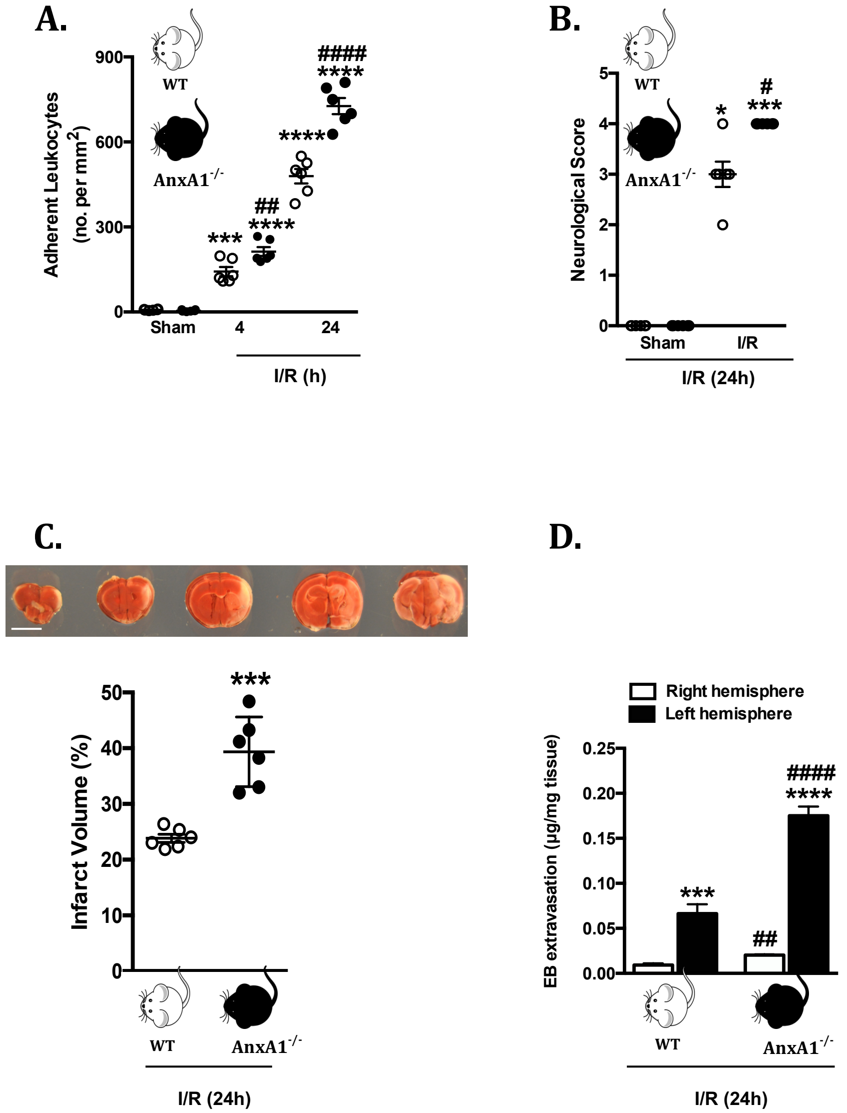

Supplemental Figure 1. AnxA1$^{-/-}$ mice display an increase in stroke severity

Wild type (WT) and AnxA1$^{-/-}$ mice were subjected to tMCAo for 60 min, followed by 4h or 24 h reperfusion. A) Intravital fluorescence microscopy was performed to assess leukocyte adhesion in the cerebral microcirculation (pial vessels) of mice subjected to cerebral ischemia reperfusion injury (I/RI). B) Neurological score (NS) was assessed. Brains were removed to quantify C) infarct volume (IV) (representative brains slices shown from WT mice. Scale bar = 5mm) and D) blood brain barrier (BBB) dysfunction was measured in right and left hemispheres from mice using Evan's blue (EB) dye extraction. Data are mean ± SEM of 6 mice per group and assessed by

ANOVA with Bonferroni post-hoc test (A+D), Kruskal-Wallis with Dunn's test (B) or a Student's *t*-test (C). $^{*}p < 0.05$, $^{***}p < 0.001$, $^{****}p < 0.0001$ vs. control. $^{\#}p < 0.05$, $^{\#\#}p < 0.01$. $^{\#\#\#\#}p < 0.0001$ vs. genotype at same time point.

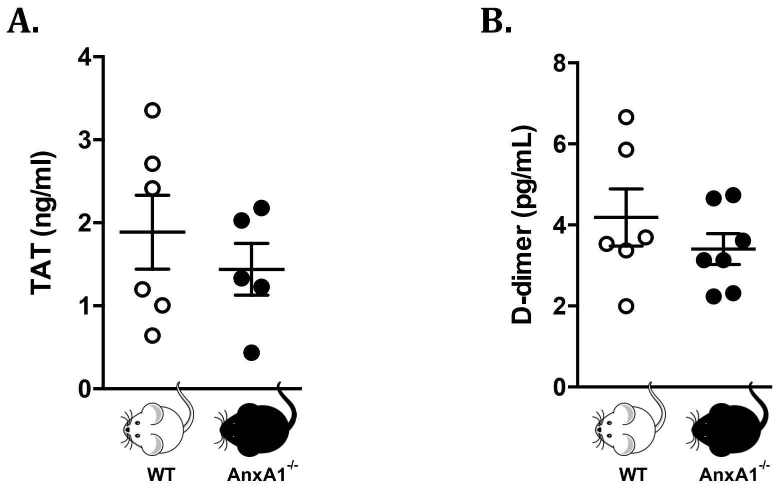

Supplemental Figure 2. Hemostatic markers are not altered in AnxA1$^{-/-}$ mice.

A) Thrombin-antithrombin (TAT) complex and B) D-dimer were measured in both WT and AnxA1$^{-/-}$ mice. Data are mean ± SEM of 5-6 mice per group.

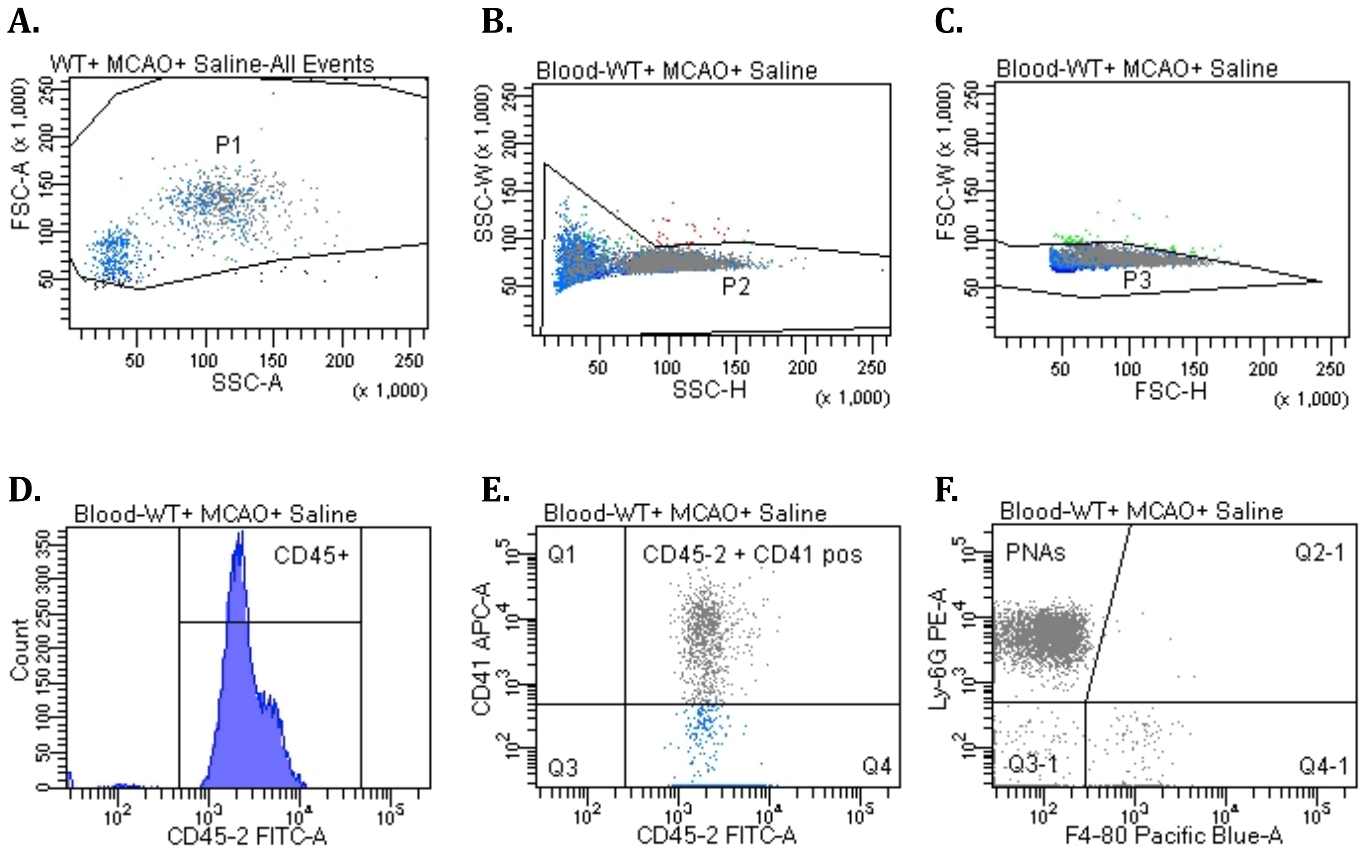

Supplemental Figure 3. Flow cytometry scattergrams illustrating the quantification of platelet-neutrophil aggregates (PNAs) from saline treated stroke mice.

Wild type (WT) mice were subjected to tMCAo for 60 min, followed 24 h reperfusion. Vehicle (saline), was administered i.v. at the start of reperfusion. Blood was taken and aggregates were quantified by flow cytometry. Representative flow cytometry scattergrams show A-C) forward scatter (size) vs. side scatter (granularity) gating for doublet discrimination and focusing on leukocyte population (P3). D) CD45+ cells based on P3 gating. E) CD45-2 + CD41 population (LPAs) gated on CD45+ cells and F) platelet-leukocyte aggregate population gated on Figure E: NPAs (Ly6G+/F4-80-), platelet-lymphocyte aggregates (Q3-1) and platelet-monocyte aggregates (Q4-1).

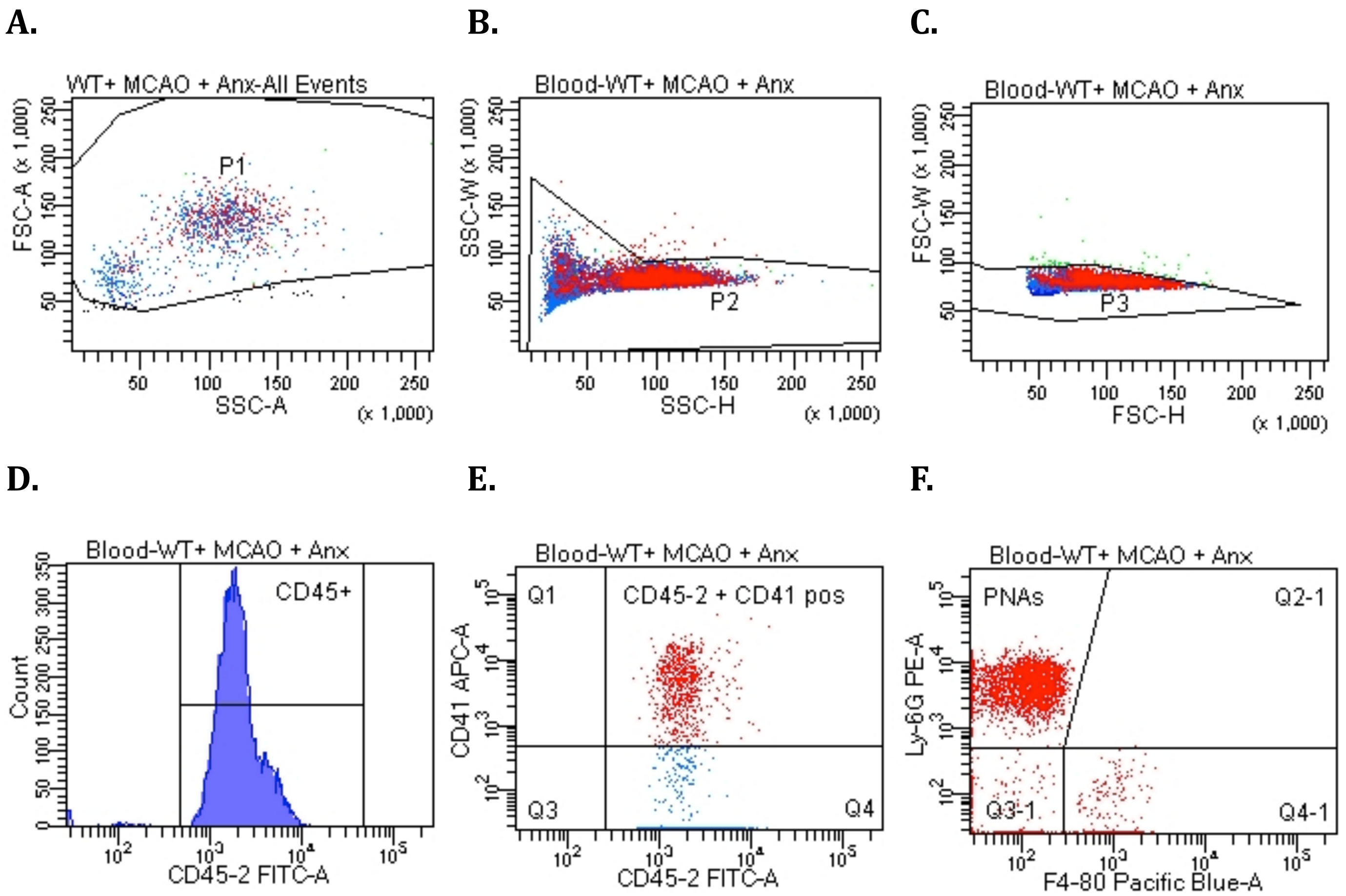

Supplemental Figure 4. Flow cytometry scattergrams illustrating the quantification of platelet-neutrophil aggregates (PNAs) from AnxA1 treated stroke mice.

Wild type (WT) mice were subjected to tMCAo for 60 min, followed 24 h reperfusion. AnxA1 (1 µg/mouse) was administered i.v. at the start of reperfusion. Blood was taken and NPAs quantified by flow cytometry. Representative flow cytometry scattergrams show A-C) forward scatter (size) vs. side scatter (granularity) gating for doublet discrimination and focusing on leukocyte population (P3). D) CD45+ cells based on P3 gating. E) CD45-2 + CD41 population (LPAs) gated on CD45+ cells and F) platelet-leukocyte aggregate population gated on Figure E: NPAs (Ly6G+/F4-80-), platelet-lymphocyte aggregates (Q3-1) and platelet-monocyte aggregates (Q4-1).

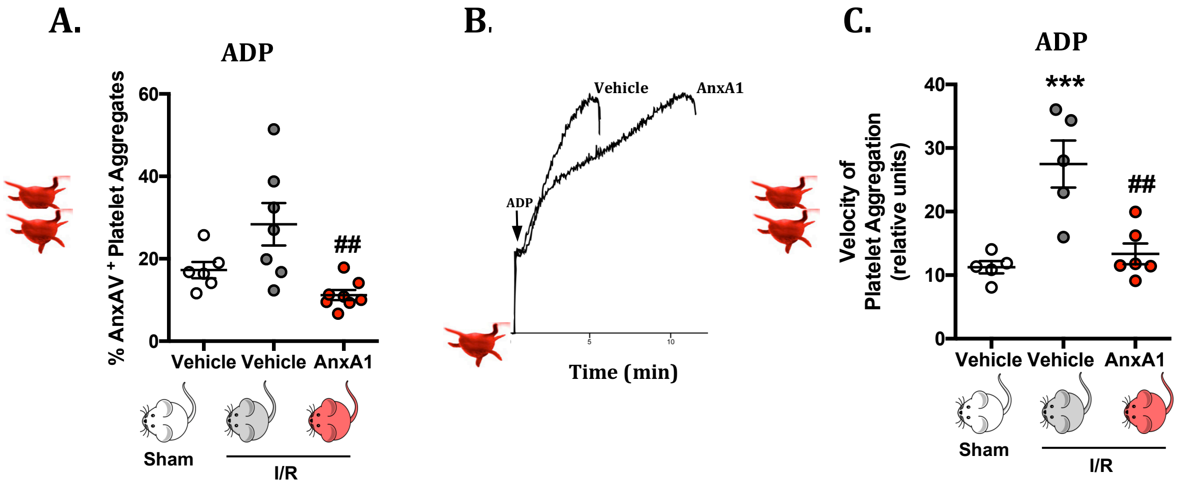

Supplemental Figure 5. AnxA1 reduces platelet activation/aggregation post ischemia reperfusion injury (I/RI).

Wild type (WT) mice were subjected to sham or transient middle cerebral artery occlusion for 60 minutes, followed by 24 h reperfusion. Vehicle (saline) or AnxA1 (3.3 mg/kg) was administered i.v. at the start of reperfusion. At the end of reperfusion, platelets were isolated for quantification. A) Using flow cytometry, phosphatidylserine (PS) externalization (using AnxAV antibody. Measured as a % of AnxAV positive platelets) was quantified on platelet-platelet aggregates following further stimulation with ADP (1 μM). B) Representative platelet aggregation velocity chart in response to ADP (1 μM). C) Velocity of aggregate formation was measured using low angle light scattering technique. Data are mean ± SEM of 5-8 mice per group and assessed by ANOVA with Bonferroni post-hoc test (A+C). $^{***}p < 0.001$ vs. sham mice. $^{\#\#}p < 0.01$ and vs. vehicle treated control I/RI mice. Scale bar 10 μm.

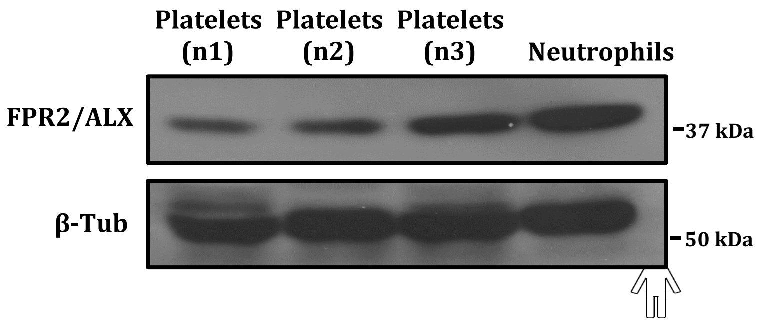

Supplemental Figure 6. Human platelets express FPR2/ALX.

Human platelets express Fpr2/ALX. Platelets were isolated and lysed with Laemmli buffer and lysates immunoblotted for FPR2/ALX and β-tubulin (neutrophil sample used as a positive control). n=3 independent donors.

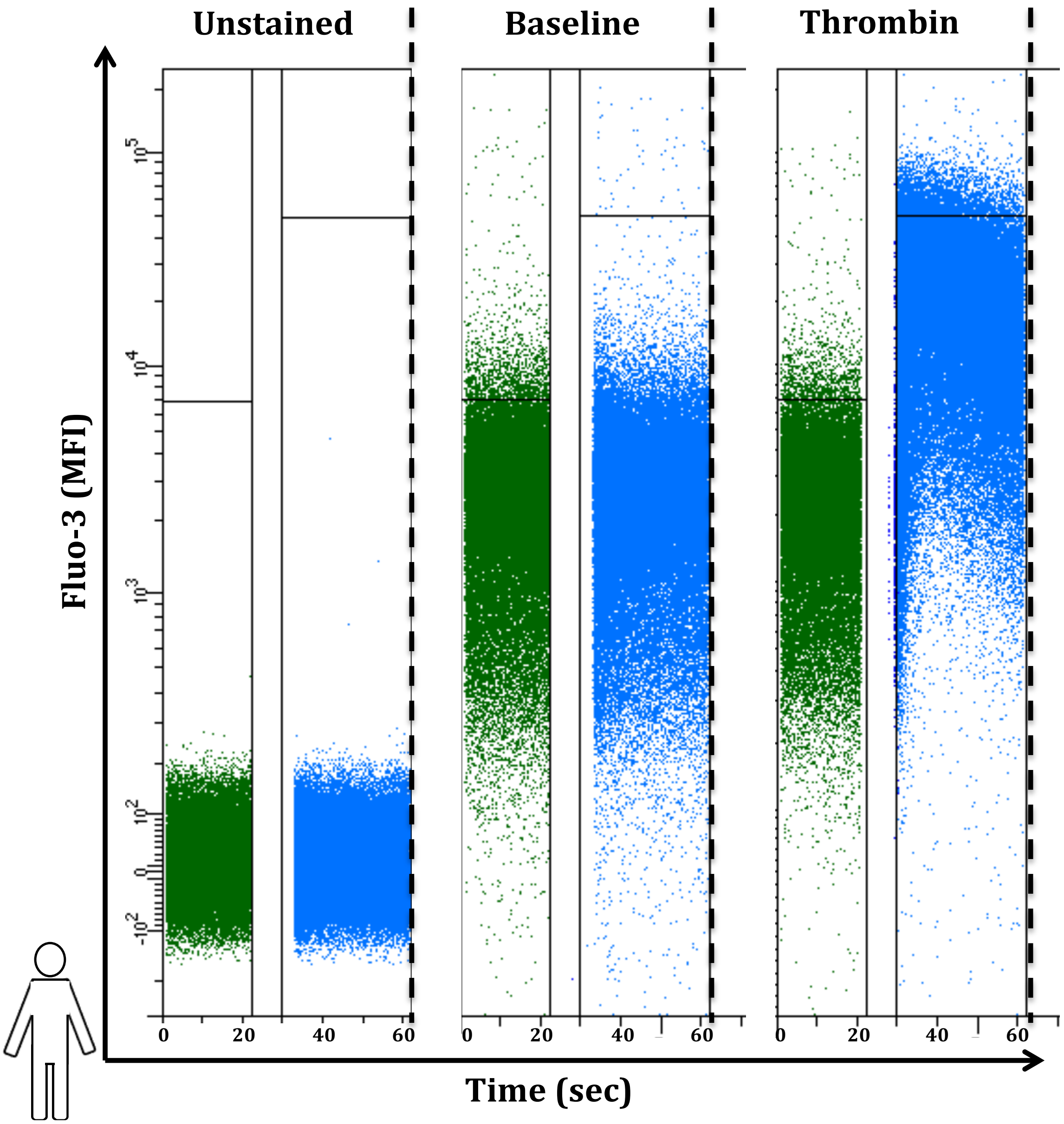

Supplemental Figure 7. Annexin 1 reduces Ca^{2+} intracellular mobilization inhuman platelets. Kinetic assay of Ca^{2+} intracellular mobilization using Real-time measure fluorescence of Fluo-3 AM loaded platelets. Baseline fluorescence of Fluo-3 AM (5 µM) was determined, followed by a pause for the addition of agonist (thrombin. 0.1U/ $1x10^6$ human platelets), after which the acquisition was resumed and the signal recorded for additional 20 s. The vertical lines crossing the

plot are regions defined on the time axis (0-20 s, 20-40 s [pause] and 40-60 sec) and dotted lines define the end of run. The picture corresponds to the populations of unstained (no Fluo-3 AM) platelets, platelets loaded with Fluo-3 AM (baseline) and thrombin-stimulated platelets (loaded with Fluo-3 AM).

Supplemental References

1. Gavins FNE, Dalli J, Flower R. Granger DN, Perretti M. Activation of the annexin 1 counter-regulatory circuit affords protection in the mouse brain microcirculation. *FASEB J* 2007;21: 1751-1758.
2. Smith HK, Gil CD, Oliani SM, Gavins FN. Targeting formyl peptide receptor 2 reduces leukocyte-endothelial interactions in a murine model of stroke. *FASEB J.* 2015;29:2161-2171.
3. Vital SA, Becker F, Holloway PM, Russell J, Perretti M, Granger DN, Gavins FN. Formyl-Peptide Receptor 2/3/Lipoxin A4 Receptor Regulates Neutrophil-Platelet Aggregation and Attenuates Cerebral Inflammation: Impact for Therapy in Cardiovascular Disease. *Circulation.* 2016;133:2169-2179.
4. Daley JM, Thomay AA, Connolly MD, Reichner JS, Albina JE. Use of Ly6G-specific monoclonal antibody to deplete neutrophils in mice. *J Leukoc Biol.* 2008;83:64-70.
5. Swamydas M, Lionakis MS. Isolation, purification and labeling of mouse bone marrow neutrophils for functional studies and adoptive transfer experiments. *J Vis Exp.* 2013;77:e50586.
6. Gavins FN, Russell J, Senchenkova EL, De Almeida Paula L, Damazo AS, Esmon CT, Kirchhofer D, Hebbel RP, Granger DN. Mechanisms of enhanced thrombus formation in cerebral microvessels of mice expressing hemoglobin-S. *Blood.* 2011;117:4125-4133.
7. Hughes EL, Becker F, Flower RJ, Buckingham JC, Gavins FNE. Mast cells mediate early neutrophil recruitment and exhibit anti-inflammatory properties *via* the formyl peptide receptor 2/lipoxin A_4 receptor. *Br J Pharmacol.* 2017;174:2393-2408.
8. Yan SL, Russell J, Harris NR, Senchenkova EY, Yildirim A, Granger DN. Platelet abnormalities during colonic inflammation. *Inflamm Bowel Dis.* 2013;19:1245-1253.

9. Mindukshev I, Gambaryan S, Kehrer L, Schuetz C, Kobsar A, Rukoyatkina N, Nikolaev VO, Krivchenko A, Watson SP, Walter U, Geiger J. Low angle light scattering analysis: a novel quantitative method for functional characterization of human and murine platelet receptors. *Clin Chem Lab Med.* 2012;50:1253-1262.

10. do Céu Monteiro M, Sansonetty F, Gonçalves MJ, O'Connor JE. Flow cytometric kinetic assay of calcium mobilization in whole blood platelets using Fluo-3 and CD41. *Cytometry.* 1999;35:302-310.

11. Yang D, Shao J, Hu R, Chen H, Xie P, Liu C. Angiotensin II promotes the anticoagulant effects of rivaroxaban via angiotensin type 2 receptor signaling in mice. *Sci Rep.* 2017;7:369.
